# An Enzymatic Strategy for the Selective Methylation of High-Value-Added Tetrahydroprotoberberine Alkaloids

**DOI:** 10.3390/ijms242015214

**Published:** 2023-10-16

**Authors:** Wanli Zhao, Manyu Liu, Kemeng Liu, Hanqing Liu, Xiufeng Liu, Jihua Liu

**Affiliations:** 1 Jiangsu Key Laboratory of TCM Evaluation and Translational Research, School of Traditional Chinese Pharmacy, China Pharmaceutical University, Nanjing 211198, China; zhaowanlitcm@126.com (W.Z.);; 2 Jiangsu Key Laboratory for the Research and Utilization of Plant Resources, Institute of Botany, Jiangsu Province and Chinese Academy of Sciences, Nanjing 210014, China; 3 State Key Laboratory of Natural Medicines, China Pharmaceutical University, Nanjing 211198, China

**Keywords:** enzymatic catalysis, biotransformation, selective methylation, tetrahydroprotoberberine alkaloids, methyltransferases

## Abstract

Tetrahydroprotoberberines (THPBs) are plant-specific alkaloids with significant medicinal value. They are present in trace amounts in plants and are difficult to chemically synthesize due to stereoselectivity and an unfavorable environment. In this study, a selective methylation strategy was developed for the biocatalysis of seven high-value-added THPB compounds using 4’-*O*-methyltransferase (*Cj4’OMT*), norcoclaurine 6-*O*-methyltransferase (*Cj6OMT*), and (*S*)-scoulerine 9-*O*-methyltransferase (*SiSOMT* and *PsSOMT*) in engineered *E. coli*. The methyltransferases *Cj4’OMT*, *Cj6OMT*, *PsSOMT*, and *SiSOMT* were expressed heterologously in *E. coli*. Compound **1** (10-methoxy-2,3,9-tetrahydroxyberbine) was synthesized using the recombinant *E. coli* strain *Cj4’OMT* and the substrate 2,3,9,10-tetrahydroxyberbine. Compound **2** (9-methoxy-2,3,10-tetrahydroxyberbine) was produced in the recombinant *Escherichia coli* (*E. coli*) strain *PsSOMT*, and compounds **2** and **3** (discretamine) were produced in the recombinant *E. coli* strain *SiSOMT*. Compounds **4** (9,10-methoxy-2,3-tetrahydroxyberbine) and **5** (corypalmine) were obtained by co-culturing the recombinant strains *Cj4’OMT* and *SiSOMT* with substrate. Compounds **6** (scoulerine) and **7** (isoscoulerine) were produced by co-culturing the substrate with the recombinant strains *Cj4’OMT* and *Cj6OMT*. To increase the yield of novel compound **2**, the flask culture conditions of the engineered *SiSOMT* strain were optimized, resulting in the production of 165.74 mg/L of this compound. This study thus presents an enzymatic approach to the synthesis of high-value-added THPBs with minimum environmental wastage.

## 1. Introduction

**Figure 1:**
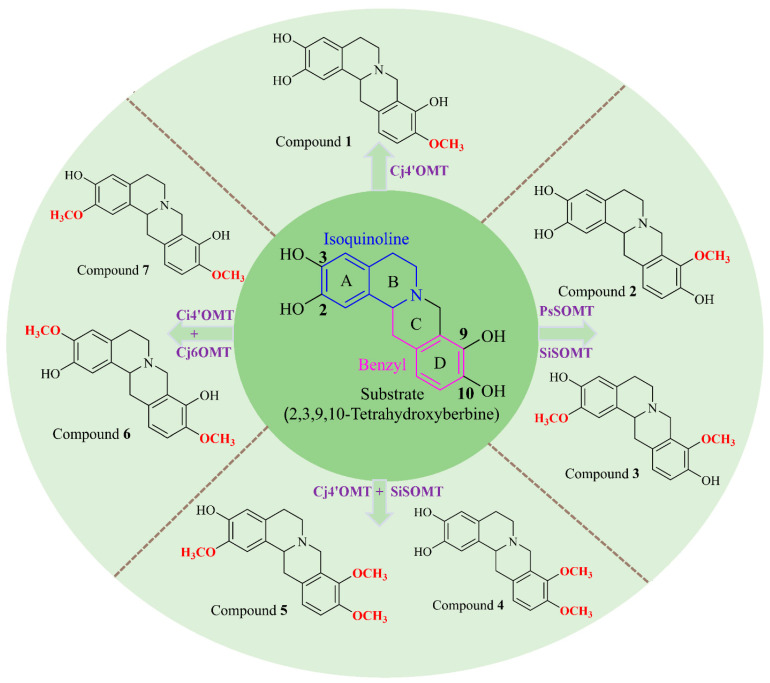
Proposed biocatalytic route from 2,3,9,10-tetrahydroxyberbine to the seven target compounds (compounds **1**, **2**, **3**, **4**, **5**, **6**, and **7**). Abbreviations: *Cj4’OMT*, 4’-*O*-methyltransferase from *Coptis japonica* (GenBank accession no. D29812); *SiSOMT*, (*S*)-scoulerine 9-*O*-methyltransferase from *Stephania intermedia* (MK749415); *PsSOMT*, (*S*)-scoulerine 9-*O*-methyltransferase from *Papaver somniferum* (JN185325); *Cj6OMT*, norcoclaurine 6-*O*-methyltransferase from *Coptis japonica* (D29811).

Tetrahydroprotoberberines (THPBs) represent a distinct category of tetracyclic alkaloids distinguished by the presence of an isoquinoline framework. The chemical structure of THPBs typically consists of methoxyl or phenolic hydroxyl groups on the A and D aromatic rings (Figure 1) [1]. The pharmacological activities of THPBs include anti-drug addiction [2], analgesia [3], anti-arrhythmia [4], and hypoglycemic effects [5]. Previous studies have shown that variations in the position and number of hydroxyl groups influence the bioactivities of THPBs [6,7,8]. THPBs are primarily extracted from plants of the *Stephania* and *Corydalis* genera, although the yields are characteristically low [9,10]. The selective methylation of phenolic hydroxyl groups on the A and D aromatic rings of THPBs during chemical synthesis requires the protection of the other phenolic hydroxyl groups [11]. These issues severely limit the supply of THPBs, restricting further investigation into their pharmaceutical utility. The use of enzymes as catalysts has significant advantages for both organic synthesis and compound yield, including chemoselectivity and enantioselectivity, together with their operation under mild conditions [12,13,14,15,16,17]. Methyltransferases are a sub-class of the transferase enzyme family and are responsible for the transfer of a methyl group from a methyl donor to an acceptor molecule [18,19]. A recent study by our group identified a methyltransferase known as SOMT (SiSOMT) derived from *Stephania intermedia*. This enzyme was shown to catalyze the synthesis of several THPB compounds [20]. Furthermore, previous research has demonstrated the involvement of the Cj4’OMT and Cj6OMT enzymes in the biosynthesis of (*S*)-reticuline, a crucial precursor in the THPB biosynthetic pathway [21,22,23]. To explore the selective methylation catalyzed by SOMT (SiSOMT, PsSOMT), Cj4’OMT, and Cj6OMT in THPB synthesis, 2,3,9,10-tetrahydroxyberbine with hydroxyl groups on C-2, C-3, C-9, and C-10 was used in this investigation.

Biosynthesis affords numerous opportunities for the industrial production of complex molecules using microbial cell factories [24,25,26]. Since it is simple to manipulate and genetic tools are widely available, *E. coli* is frequently utilized as a prokaryotic host for chemical production [27,28]. In addition, *E. coli* produces more THPB precursors and intermediates, such as L-tyrosine and reticuline, than other organisms [29,30,31,32]. In this study, seven THPB compounds were biosynthesized directly from 2,3,9,10-tetrahydroxyberbine using Cj4’OMT, Cj6OMT, PsSOMT, and SiSOMT (Figure 1). This study describes a novel method for the production of THPB-based drugs, which has significant potential for future large-scale production.

## 2. Results and Discussion

### 2.1. Heterologous Expression of Recombinant Proteins and Enzyme Assays

**Figure 2:**
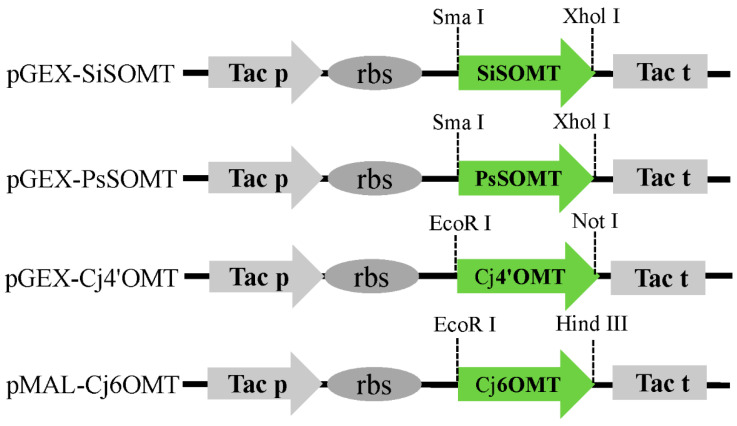
Schematic diagram of constructions with the recombinant plasmid *SiSOMT*, *PsSOMT*, *Cj4’OMT*, and *Cj6OMT*.

The feasibility of the proposed biocatalytic route (see Figure 1) was investigated by expressing the enzymes in vitro and testing their activities. The recombinant plasmids pGEX-SiSOMT, pGEX-PsSOMT, pGEX-Cj4’OMT, and pMAL-Cj6OMT were constructed and transformed into BL21 (DE3) to obtain the BL21-pGEX-SiSOMT, BL21-pGEX-PsSOMT, BL21-pGEX-Cj4’OMT, and BL21-pMAL-Cj6OMT strains, respectively (Figure 2 and Table 1). HPLC-QTOF-MS/MS was used to analyze the bioconversion products of the recombinant crude enzymes. The results showed that Cj4’OMT and SiSOMT were able to catalyze 2,3,9,10-tetrahydroxyberbine in vitro. Cj4’OMT with 2,3,9,10-tetrahydroxyberbine exhibited a peak at *m*/*z* 314.1391 ([M + H]^+^, compound **1**) along with a substrate peak at *m*/*z* 300.1236 ([M + H]^+^) (Table S2). Compound **1** had a molecular weight 14 Da higher than that of the substrate, indicating the addition of one −CH_3_ group. The C-ring cleavage from *m*/*z* 314.1391 revealed product ions at *m*/*z* 164 and *m*/*z* 151, demonstrating the presence of an additional methyl group on the D ring. SiSOMT-catalyzed 2,3,9,10-tetrahydroxyberbine yielded two peaks at *m*/*z* 314.1389 ([M + H]^+^, compound **2**) and *m*/*z* 328.1535 ([M + H]^+^, compound **3**). A comparison of the characteristic ions in compound **2** and compound **3** revealed the presence of one and two additional methyl groups relative to the substrate, respectively. SiSOMT and Cj4’OMT with 2,3,9,10-tetrahydroxyberbine showed two peaks at *m/z* 328.1532 ([M + H]^+^, compound **4**) and *m*/*z* 342.1690 ([M + H]^+^, compound **5**). Similarly, Cj4’OMT and Cj6OMT with 2,3,9,10-tetrahydroxyberbine exhibited two peaks at *m/z* 328.1546 ([M + H]^+^, compound **6**) and *m*/*z* 328.1540 ([M + H]^+^, compound **7**). Only the ion peak of the substrate was observed in the negative control samples. It was hypothesized that SiSOMT, PsSOMT, Cj4’OMT, and Cj6OMT contributed to the selective methylation of THPBs.

**Figure 3:**
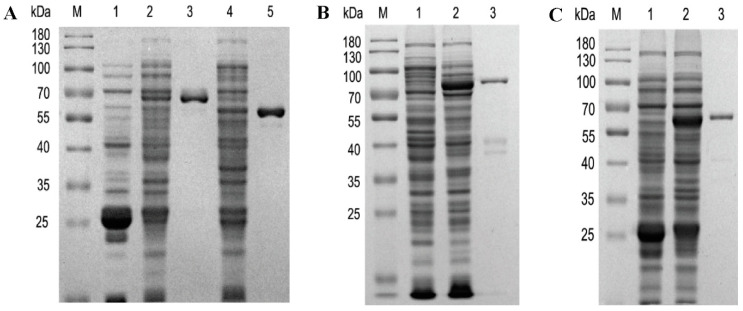
SDS-PAGE of the purified recombinant proteins. M, marker; (**A**), lane 1: control (pGEX-6p-1 vector); lane 2: crude enzyme of PsOMT; lane 3: purified PsOMT; lane 4: crude enzyme of SiSOMT; lane 5: purified SiSOMT; (**B**), lane 1: control (pMAL-c4x); lane 2: crude enzyme of Cj6OMT; lane 3, purified Cj6OMT; (**C**), lane 1: control (pGEX-6p-1 vector); lane 2: crude enzyme of Cj4’OMT; lane 3: purified Cj4’OMT.

The kinetic parameters of Cj4’OMT, Cj6OMT, PsSOMT, and SiSOMT were then investigated to examine the catalytic potential of the recombinant methyltransferases. The recombinant enzymes were purified from crude enzyme extracts using a protein purification column (SiSOMT, PsSOMT, and Cj4’OMT were purified using a GST tag while Cj6OMT was purified using an MBP tag). The purified recombinant enzymes were examined using SDS-PAGE (Figure 3). The kinetic parameters of SiSOMT, PsSOMT, Cj4’OMT, and Cj6OMT were determined using 2,3,9,10-tetrahydroxyberbine or compound **1** as the substrate and the results are presented in Figure S1. The *K_m_* of SiSOMT was found to be lower than that of PsSOMT, Cj4’OMT, and Cj6OMT, while the turnover number (*k*_cat_) of SiSOMT was 3.2-fold higher than that of PsSOMT (Table 2). The catalytic efficiency (*k*_cat_*/K*_m_) of SiSOMT was 3.9-fold higher than that of PsSOMT and 2.9-fold higher than that of Cj4’OMT. Overall, SiSOMT was found to be more efficient in 2,3,9,10-tetrahydroxyberbine methylation than Cj4’OMT and PsSOMT. 

### 2.2. Structural Elucidation of Compounds

**Figure 4:**
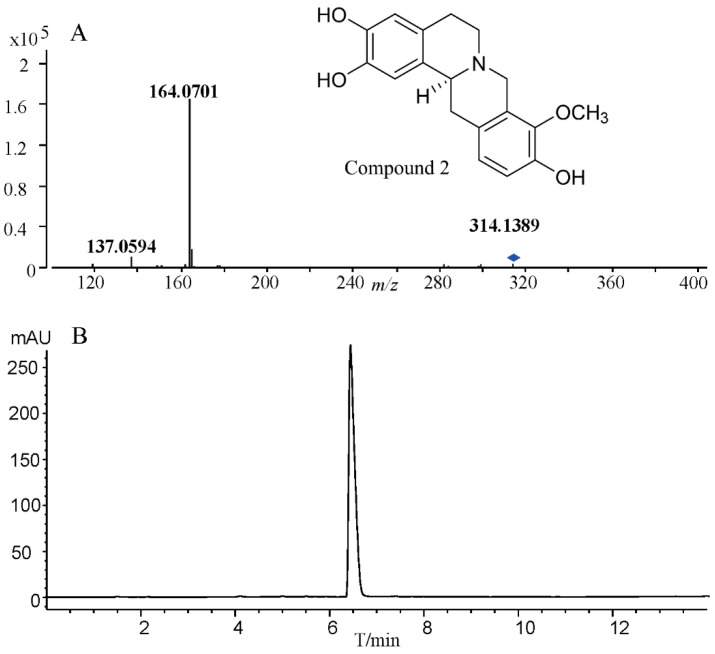
Mass spectrometry and UHPLC of compound **2**. (**A**) the exact mass of [M + H]^+^ compound **2** and (**B**) UHPLC analysis of purified compound **2** at 280 nm.

The results confirmed that the whole-cell system carrying the methyltransferase gene for the selective methylation of 2,3,9,10-tetrahydroxyberbine can produce THPBs. Compounds **1**, **2**, **3**, **4**, **5**, **6**, and **7** were biosynthesized using the whole-cell system and purified by means of preparative liquid chromatography. The structure of compounds **1**, **2**, **3**, **4**, **5**, **6**, and **7** was confirmed via NMR analysis (^1^H, ^13^C). Compound **1** (10-methoxy-2,3,9-tetrahydroxyberbine), a white powder, yielded 52%, ${\left[\alpha \right]}_{D}^{20}$ −281.90 (*c* 0.12, MeOH), 95.58% e.e. Compound **1** is consistent with the NMR data for 10-methoxy-2,3,9-tetrahydroxyberbine previously reported [3]. The ^1^H, ^13^C-NMR, and HRESIMS data for compound **1** are shown in Supporting Information S2 and Figure S2. PsSOMT has been shown to be regiospecific as 9-*O*-methyltransferases target (*S*)-scoulerine [33]. Consistent with previous findings, it was found that PsSOMT exhibited high regiospecificity as the 9-*O*-methyltransferases targeted 2,3,9,10-tetrahydroxyberbine. In contrast, SiSOMT was able to sequentially provide 9- and 2-*O*-methylate 2,3,9,10-tetrahydroxyberbine. Strain BL21-pGEX-PsSOMT (harboring the *PsSOMT* gene) was used to produce compound **2**: compound **2** (9-methoxy-2,3,10-tetrahydroxyberbine), white powder, yielded 75%, ${\left[\alpha \right]}_{D}^{20}$ −226.79 (*c* 0.22, MeOH), 99.38% e.e. The ^1^H, ^13^C-NMR COSY, HMBC, HSQC, and mass spectrometry data for compound **2** (Supporting Information S2 and Figure 4) revealed the molecular ion at *m*/*z* 314.1389 [M + H]^+^, indicating the addition of the -CH_3_ group in comparison with the substrate. The characteristic methoxymethyl signals that appeared at δ = 3.83 ppm and δ = 60.41 (-OCH_3_ group) in the ^1^H and ^13^C NMR results, respectively, indicate oxygen attachment in the C-9 or C-10 position. Compound **2** was identified as 9-methoxy-2,3,10-tetrahydroxyberbine via COSY, HMBC, and HSQC. Compound **3** was purified in two steps with the second step involving purification on a C_18_HCE column (4.6 × 150 mm, 5 μm) using isocratic elution with 15% acetonitrile. The HPLC profile (Figure S3) shows the purity of compound **3**: compound **3** (discretamine), white powder, yielded 65%, ${\left[\alpha \right]}_{D}^{20}$ −295.16 (*c* 0.12, MeOH), 100% e.e. The ^1^H and ^13^C-NMR spectra of compound **3** (Supporting Information S2) are consistent with those of discretamine reported in the literature [34]. The co-culture strategies successfully produced various compounds, including pyranoanthocyanins [35], flavonoids [36], and curcuminoids [37]. The *E. coli* co-cultures with two engineered strains were used to incorporate three methyl groups in 2,3,9,10-tetrahydroxyberbine. The recombinant *E. coli* strains BL21-pGEX-Cj4’OMT and BL21-pGEX-SiSOMT were co-cultured to synthesize compound **4** and compound **5** followed by identification using ^1^H and ^13^C-NMR (Supporting Information S2, Figures S4 and S5). Similarly, the recombinant *E. coli* strains BL21-pGEX-Cj4’OMT and BL21-pMAL-Cj6OMT were co-cultured to produce compounds **6** and **7**. Compounds **6** and **7** were isolated from the fermented broth extract using pre-HPLC and identified using ^1^H and ^13^C-NMR (Supporting Information S2, Figures S6 and S7).

### 2.3. Molecular Docking of SOMT to the Substrate

Specifically, PsSOMT exhibited strict regiospecificity as the 9-*O*-methyltransferases targeted only 2,3,9,10-tetrahydroxyberbine, while SiSOMT was able to sequentially produce 9- and 2-*O*-methylate 2,3,9,10-tetrahydroxyberbine. Molecular docking was used for elucidation of the interactions between SiSOMT and PsSOMT and the substrate. TfSOMT (PDB: 6NEG, from *Thalictrum flavum*) and PsSOMT1 (PDB: 6I6K, from *Papaver somniferum*) were used as the templates for the homology modeling of SiSOMT and PsSOMT, respectively. The results demonstrated that the active site of SiSOMT was formed by three amino acids, with Lys250 interacting with C-9 while His219 and Leu217 interacted with the C-2 of the substrate. In PsSOMT, Lys292 and Ser211 interacted with the C-9 of the substrate. This suggests that His219 and Leu217 may contribute to SiSOMT’s capacity for two successive methylations (Figure S8). 

### 2.4. Optimization of the Fermentation Conditions for Producing Compound **2**

**Figure 5:**
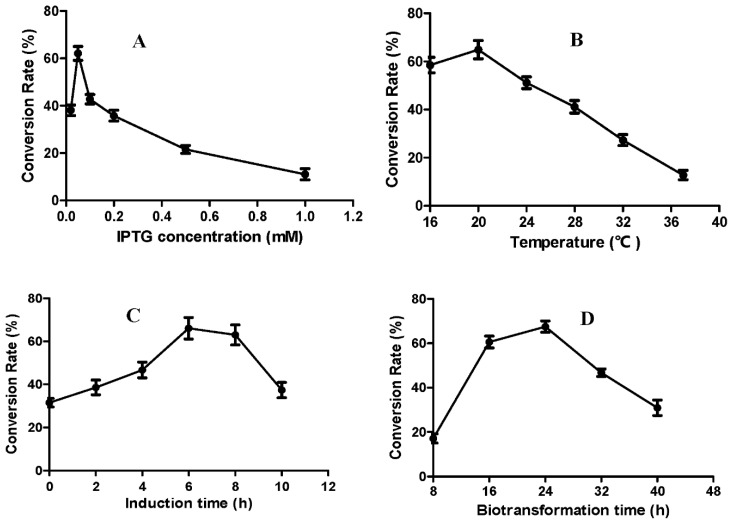
Optimization of the induction dose (**A**), induction temperature (**B**), induction time (**C**), and biotransformation time (**D**) for producing compound **2**. Error bars denote the standard deviation from three independent experiments.

The fermentation parameters of the engineered BL21-pGEX-PsSOMT strain, including different induction doses, temperatures, and times, as well as the biotransformation time, were optimized to improve the fermentation yield of compound **2** (Figure 5). The optimized procedure involved the inoculation of 1% of the seed solution into 100 mL of TB, with culturing for 3 h at 37 °C and continuous shaking at 220 rpm. Isopropyl β-D-Thiogalactoside (IPTG) was added to a final concentration of 0.05 M to induce enzyme expression. Following 6 h of induction at 20 °C, 20 mg of substrate was added and incubated for 24 h. Under these optimal conditions, a flask culture was capable of producing 165.74 mg/L (conversion rate 82.87% ± 3.79) of compound **2**, four-fold higher than the yield obtained before optimization. 

In this study, an enzymatic strategy was established for the selective methylation of alkaloids using either single methyltransferases or a co-culture approach for the directed methylation of seven high-value-added THPBs. THPBs are plant-specific alkaloids with potent activity. For example, compound **3** has been shown to have anti-inflammatory effects [38] and compound **5** has been reported to exhibit antibacterial activity [39], while, compound **6** shows anti-tumor activities [6]. These data demonstrated that the methylation of THPBs at various locations resulted in a wide range of activities. Therefore, a synthetic approach requires further refinement to achieve an increased yield of THPBs. To further investigate the physiological processes mediated by this selective methylation, we intend to construct a library of methylated THPBs. The kinetic parameters of Cj4’OMT, Cj6OMT, PsSOMT, and SiSOMT were investigated to explore the catalytic potential of the recombinant methyltransferases. The results demonstrated that the different methyltransferases showed varying affinities for the substrate. For example, the *Km* values of PsSOMT, SiSOMT, and Cj4’OMT using 2,3,9,10-tetrahydroxyberbine as the substrate were observed to be 53.77 ± 12.03, 44.09 ± 10.91, and 83.50 ± 19.72, respectively. Furthermore, the SOMTs derived from *S. intermedia* and *P. somniferum* showed distinct catalytic efficiencies (SiSOMT exhibited higher catalytic efficiency than PsSOMT). The kinetic parameters of PsSOMT for the catalysis of 2,3,9,10-tetrahydroxyberbine differed from those reported previously for scoulerine [33]. It was speculated that the methoxy groups at positions C-3 and C-10 on scoulerine alter the enzyme affinity and, consequently, the catalytic efficiency. This suggests that site-specific improvements could be conducted on these methyltransferases to increase their activity for the targeted biosynthesis of THPB compounds. In addition, *E. coli*-produced SAM was used as the methyl donor in the present study. It is possible that S-adenosyl-L-methionine (SAM) may become a limiting factor for substrate conversion with increased substrate concentrations. Genetically engineered *E. coli* could be used to produce more SAM as a methyl donor for fermentation-scale supply. 

THPBs typically have an isoquinoline structure with a four-ring backbone. The critical positions for the functional groups are C-2, C-3, C-9, and C-10 [40]. Differential combinations of these functional groupings result in a wide range of therapeutic potential [41]. However, the majority of THPBs are purified from plants with characteristically low yields, and chemical synthesis is expensive and associated with stereochemical challenges. This research described an efficient approach to synthesizing THPBs, resulting in the discovery of a novel compound. It is essential to evaluate the structure–activity relationships of these compounds in our follow-up research. Methyltransferases remain one of the most promising biocatalysts in green organic synthesis because of their excellent catalytic potential involving shorter routes without the need for protection steps, less environmentally detrimental waste, and lower equipment costs and energy use. These advantages can lead to efficient and practical pharmaceutical production that is significantly better than conventional chemical routes. The findings of this study demonstrate an efficient strategy for the direct production of THPBs from 2,3,9,10-tetrahydroxyberbine via whole-cell biocatalysis in *E. coli*. To the best of our knowledge, no previous studies have demonstrated the directional methylation of THPBs without the formation of side products. Accordingly, this study represents a significant milestone toward the development of green THPB synthesis. As demonstrated earlier, this method is effective and simple, and it has the potential for large-scale application. 

## 3. Materials and Methods

### 3.1. Materials, Reagents, and Instruments

All bacterial strains and plasmids used in the study are listed in Table 1. The *E. coli* strains were routinely cultured in lysogeny broth (LB) supplemented with 100 mg/L ampicillin. The engineered strains were cultured in terrific broth (TB) to produce alkaloid products. All the chemicals were purchased from TEDIA (Tedia Co., Fairfield, OH, USA). The structure of the synthesized 2,3,9,10-tetrahydroxyberbine hydrochloride was confirmed using ^1^H- and ^13^C-NMR. The primers were synthesized by Tsingke (Nanjing, China). The PCR polymerases and DNA ligases were purchased from Vazyme (Nanjing, China). Optical rotations were recorded with a JASCO P-1020 polarimeter (JASCO, Tokyo, Japan). The NMR experiments (^1^H: 500 MHz, ^13^C: 125 MHz) were measured using a Bruker AVIII-500 NMR instrument (Bruker, Bremen, Germany) equipped with a broadband observe probe. TMS was used as an internal standard, and either MeOD or CDC_l3_ was used as solvent for dissolving compounds. 

### 3.2. Plasmid Construction

The recombinant plasmids were constructed as previously described with minor modifications [42]. *SiSOMT* (MK749415) was obtained using PCR amplification and cloned into pGEX-6p-1 (Sma I/Xhol I) to generate recombinant plasmid pGEX-SiSOMT. The recombinant plasmids pGEX-PsOMT and pGEX-Cj4’OMT were obtained by cloning *PsOMT* (JN185325) and *Cj4’OMT* (D29812), respectively, into pGEX-6p-1 (Sma I/Xhol I), while pMAL-Cj6OMT was obtained by cloning *Cj6OMT* (D29811) into pMAL-c4x. The primers used for plasmid construction are listed in Supporting Information S1 (Table S1). The constructed plasmids were identified using colony PCR and Sanger sequencing before introduction into BL21 (DE3) for protein expression.

### 3.3. Heterologous Expression of Recombinant Proteins in E. coli

The *E. coli* BL21 (DE3) containing the recombinant plasmids was pre-cultured in 5 mL of LB (liquid media) supplemented with t appropriate antibiotics and incubated overnight at 37 °C. A volume of 0.5 mL of the seed broth was cultured in 50 mL of TB at 37 °C for 3 h, before induction by the addition of 0.1 mM IPTG, and was incubated for an additional 18 h at 16 °C. After centrifugation (6000× *g*, 3 min, 4 °C), the cells were collected and resuspended in binding buffer. The suspension was homogenized for 15 min using a sonicator, followed by the removal of the cell debris using centrifugation at 6000× *g* for 10 min. The supernatant containing the crude enzymes was collected and the enzymes were purified using a protein purification column. The enzyme activities of SiSOMT, PsSOMT, Cj4’OMT, and Cj6OMT were assayed using a reaction mixture of 100 μL of buffer (100 mM Tris-HCl) and 0.4 mg of pure recombinant enzyme. Varying substrate concentrations ranging from 50 to 500 μM of 2,3,9,10-tetrahydroxyberbine were incubated at 37 °C for 2–32 min in 100 mM Tris-HCl. UHPLC was used to quantify the bioconversion products.

### 3.4. In Vitro Screening of Selective Methylation by the Methyltransferases

Selective methylation by SiSOMT, PsSOMT, Cj4’OMT, and Cj6OMT was investigated using 2,3,9,10-tetrahydroxyberbine as the substrate. Assays of methylation activity were performed in 100 mM Tris-HCl with 0.5 mM of substrate, 5 mM of S-adenosyl methionine (SAM), and 100 μL of crude enzyme. The negative controls included the same reagents, with the exception that the crude enzyme was boiled for 10 min. After incubating the mixtures for 18 h at 37 °C, the reaction was quenched by the addition of 100 μL of methanol. The products were identified using HPLC-QTOF-MS/MS.

### 3.5. Whole-Cell Biotransformation for Compound Production

The *E. coli* strain BL21-pGEX-Cj4’OMT was grown in 50 mL of TB for 3 h at 37 °C and shaken at 220 rpm for the production of compound **1** by whole-cell biotransformation. Then, 0.1 mM of IPTG was added to 50 mL of TB, which was incubated at 18 °C for 8 h. Biosynthesis was carried out with 20 mg of 2,3,9,10-tetrahydroxyberbine for 24 h at 20 °C. The BL21-pGEX-PsSOMT and BL21-pGEX-SiSOMT strains were grown in 50 mL of TB for 3 h at 37 °C and shaken at 220 rpm to produce compounds **2** and **3**, respectively. IPTG (0.1 mM) was then added and the *E. coli* cells were cultured for 8 h at 16 °C. For biosynthesis, 20 mg of 2,3,9,10-tetrahydroxyberbine was added and incubated for 24 h at 16 °C and shaken at 220 rpm. The BL21-pGEX-Cj4’OMT and BL21-pGEX-SiSOMT strains were each cultured for 3 h in 50 mL of TB at 37 °C. After the addition of 0.1 mM of IPTG, the mixture was incubated for 12 h at 16 °C and shaken at 220 rpm. The reaction of 20 mg of 2,3,9,10-tetrahydroxyberbine with BL21-pGEX-Cj4’OMT and BL21-pGEX-SiSOMT at 20 °C for 24 h yielded compounds **4** and **5**. Strain BL21-pGEX-Cj4’OMT was grown in TB (50 mL) at 37 °C (220 rpm) for 3 h to produce compounds **6** and **7**. Following the addition of 0.1 mM of IPTG, the mixture was incubated for 8 h at 16 °C. The biosynthetic reaction with 20 mg of 2,3,9,10-tetrahydroxyberbine was conducted for 24 h at 20 °C and shaken at 220 rpm. The culture broth was extracted using twice the volume of ethyl acetate. Following the evaporation of the organic solvent, the products were dissolved in methanol. The strain BL21-pMAL-Cj6OMT was grown in 50 mL of TB for 3 h at 37 °C and 220 rpm. After the addition of 0.1 mM of IPTG, the mixture was incubated for 8 h at 16 °C. The biosynthetic reaction with the strain BL21-pGEX-Cj4’OMT products was conducted for 24 h at 20 °C and shaken at 220 rpm. After biotransformation, the culture broth was extracted with two volumes of ethyl acetate before the evaporation of the organic solvent. The products were suspended in methanol for analysis and separation.

### 3.6. Product Analysis 

The bioconversion products from the recombinant *E. coli* strains were analyzed using a 1260 HPLC system coupled to a 6530 quadrupole time-of-flight mass spectrometer. The thermal column compartment was equipped with a ZORBAX C_18_ column. The mobile phase A consisted of a solution containing 0.1% formic acid and 1 mM of ammonium formate in water, whereas the mobile phase B was composed of acetonitrile. The following linear gradient program was used: 15% B for 0–5 min, 15–20% B for 5–10 min, 20–22% B for 10–15 min, 22% B for 15–17 min, 22–30% B for 17–20 min, 30–95% B for 20–22 min, 95–15% B for 22–23 min, and 15% B for 23–25 min. The flow rate of the mobile phase was set to 1 mL min^−1^ with an injection volume of 5 μL, and detection was carried out at 280 nm. The mass spectra were acquired in the positive ion mode with the following acquisition parameters: 4000 V capillary voltage; 35 psi nebulizer pressure; 10.0 L min^−1^ drying gas flow rate; 350 °C drying gas temperature; and 120V fragmentor voltage. Accurate mass spectra were recorded over the range of 100–1500 *m*/*z*, and collision energies ranged between 10 and 50 eV. The tolerance of mass accuracy for the compound searches and formula generation was set to ±5 ppm.

The compounds were quantitatively analyzed to calculate the conversion rate using an Agilent 1290 UHPLC (Santa Clara, CA, USA). Chromatographic separation was performed on an Agilent Ec-C_18_ column at 28 °C. Water (containing 0.1% formic acid and 1 mM of ammonium formate; phase A) and acetonitrile (phase B) were used as the mobile phase. The separation gradient was as follows: 5% B, 0–1.0 min; 5–15% B, 1.0–3.5 min; 15% B, 3.5–4.0 min; 15–25% B, 4.0–5.5 min; 25–35% B, 5.5–8.0 min; 35% B, 8.0–9.0 min; 35–95% B, 9.0–12 min; 95–5.0% B, 12–13 min; and 5% B, 13–14 min. The detection wavelength was set to 280 nm, and the injection volume and flow rate were 3 µL and 0.2 mL min^−1^, respectively.

### 3.7. Product Separation and Structural Analysis

The culture broth was extracted in two volumes of ethyl acetate. The organic phase was recovered and dried via the evaporation of the organic solvent. The remaining products were dissolved in methanol, and the crude extracts were then extracted from the supernatant. Two purification methods were used for further purification of the crude extracts. First, the crude compounds were chromatographed using prep-HPLC under the following conditions: column, flash; UV detection, 280 nm and 254 nm; gradient elution of MeOH (A)–Dichloromethane (B): flow rate of 20 mL min^−1^, 0% (A, 0–3.0 min), 0–5% (A, 3.0–5.0 min), 5% (A, 5.0–6.0 min), 5–10% (A, 6.0–8.0 min), 10% (A, 8.0–11.0 min), 10–20% (A, 11.0–13.0 min), 20% (A, 13.0–16.0 min), 20–40% (A, 16–19 min), and 40% (A, 19–23 min). The fractions were then purified using prep-HPLC (Agilent 1100) as follows: column ODS-C_18_ (250 mm × 20 mm × 5 μm, Hedera, China); gradient elution of water with 0.1% formic acid (A)–acetonitrile (B): 10% (B, 0–3.0 min), 10–25% (B, 3.0–7.0 min), 25–28% (B, 7.0–10.0 min), 28% (B, 10.0–13.0 min), 28–90% (B, 13.0–13.5 min), 90–10% (B, 13.4–14.0 min), and 10% (B, 14.0–15.0 min) with a flow rate of 3 mL min^−1^ and detecting wavelengths of 280 and 254 nm. Various spectral techniques were utilized to determine the structures of the purified compounds. The structures of the purified compounds were confirmed using NMR and HRESIMS. In addition, the chemical shifts of the hydrogens in compound **2** were assigned using correlation spectroscopy (COSY), heteronuclear multiple-bond correlation spectroscopy (HMBC), and heteronuclear single quantum coherence (HSQC).

## Figures and Tables

**Table 1:** Plasmids and strains used in this study.

Plasmids/Strains	Detailed Information	Source/Usge
pGEX-PsSOMT	pGEX-6p-1-Tac Prom-PsSOMT-Tac Term, Sma I/Xhol I	This work
pGEX-SiSOMT	pGEX-6p-1-Tac Prom-SiSOMT-Tac Term, Sma I/Xhol I	This work
pGEX-Cj4’OMT	pGEX-6p-1-Tac Prom-Cj4’OMT-Tac Term, EcoR I/Not I	This work
pMAL-Cj6OMT	pMAL-c4x, Tac Prom-Cj6OMT-Tac Term, EcoR I/Hind III	This work
*E. coli* DH5α	F-φ80 lacZΔM15Δ (lacZYA-argF) U169 end A1 recA1 hsdR17 (rk-, mk-) supE44λ- thi-1 gyrA96 relA1 phoA	Plasmid amplification
*E. coli* BL21(DE3)	F-ompT hsdS (rB-mB-) gal dcm (DE3)	Protein expression
BL21-pGEX-PsSOMT	*E. coli* BL21(DE3) with pGEX-PsSOMT	Producing alkaliods
BL21-pGEX-SiSOMT	*E. coli* BL21(DE3) with pGEX-SiSOMT	Producing alkaliods
BL21-pGEX-Cj4’OMT	*E. coli* BL21(DE3) with pGEX-Cj4’OMT	Producing alkaliods
BL21-pMAL-Cj6OMT	*E. coli* BL21(DE3) with pMAL-Cj6OMT	Producing alkaliods

**Table 2:** The kinetic parameters of PsSOMT, SiSOMT, Cj4’OMT, and Cj6OMT.

Enzyme	Substrate	*K_m_* (μΜ)	Vmax nmol min^−1^ mg^−1^ Protein	*k*_cat_ (S^−1^)	*k*_cat_/*K*_m_ (M^−1^ S^−1^)
PsSOMT	2,3,9,10-tetrahydroxyberbine	53.77 ± 12.03	522 ± 27	0.52 ± 0.03	9671
SiSOMT	2,3,9,10-tetrahydroxyberbine	44.09 ± 10.91	1682 ± 92	1.68 ± 0.09	38,149
Cj4’OMT	2,3,9,10-tetrahydroxyberbine	83.50 ± 19.72	1088 ± 79	1.09 ± 0.08	13,018
Cj6OMT	compound **1**	59.46 ± 15.98	505 ± 36	0.13 ± 0.01	2186

## Data Availability

The datasets generated during and/or analyzed during the current study are available from the corresponding authors on reasonable request.

## Supplementary Materials

The following supporting information can be downloaded at https://www.mdpi.com/article/10.3390/ijms242015214/s1.
