# Supplementary material for: An Enzymatic Strategy for the Selective Methylation of High-Value-Added Tetrahydroprotoberberine Alkaloids
Source: Int J Mol Sci. 2023 Oct 16;24(20):15214. doi: 10.3390/ijms242015214 (PMC10607743; doi:10.3390/ijms242015214)
Supplement: Supplementary file 1 [file ijms-24-15214-s001.zip › ijms-2628782-supplementary.pdf]

Supporting Information S1

Table S1 the primers used for plasmid construction.

Primer	Oligonucleotide sequences (5'→3')	Restriction
SiSOMT-F	ggatccccggaattcccgggGAAGGTGCCAACTTCCTAGCAG	Sma I / XhoI I
SiSOMT-R	tcacgatgcggccgctcgagCTTTAGGAATTCCAAGACATGAAGG	
PsSOMT-F	ggatccccggaattcccgggATGGTAAAAAAGATGGAAGTAGTAAGTAAGA	Sma I / XhoI I
PsSOMT-R	atgcggccgctcgagtcgacTCAATGAGGGTAAGCCTCAATAACA	
6P-S-X-F	GTCGACTCGAGCGGCCGC	
6P- S-X-R	CCCGGGAATTCCGGGGAT	
4'OMT-F	ccctgggatccccggaattcATGTCTTTCCATGGGAAAGATGA	EcoR I / Not I
4'OMT-R	gtcagtcacgatgcggccgcCTATGGAAAAACCTCAATGACTGATT	
6P-E-N-F	GCGGCCGCATCGTGACTG	
6P-E-N-R	GAATTCCGGGGATCCCAGG	
6OMT-F	agggaaggatttcagaattcGAATTCATGGAAGTGAAGAAGGACA	EcoR I / Hind III
6OMT-R	cgacggccagtgccaagcttAAGCTTCTAGTGGTGGTGGTGG	
M-E-H-F	AAGCTTGGCACTGGCCGT	
M-E-H-R	GAATTCTGAAATCCTTCCCTCGA	

Note: 6P-S-X, 6P-E-N, M-E-H: the primer of vector; lowercase: the homologous sequences of gene and vector.

Table S2 HPLC-QTOF-MS/MS data for the products

**Enzyme**	**Substrate**	**t_R (min)**	**$[M+H]^+$**	**Error (ppm)**	**Fragment ions**	**Formula**	**Identification**
Cj4'OMT	(*S*)-2,3,9,10-tetrahydroxyberbine	9.60	300.1227	1.12	191.0853, 164.0704, 137.0595	$C_{17}H_{17}NO_4$	(*S*)-2,3,9,10-tetrahydroxyberbine
		12.20	314.1391	-1.33	164.0701, 151.0761, 137.0591	$C_{18}H_{19}NO_4$	Compound **1**
SiSOMT	(*S*)-2,3,9,10-tetrahydroxyberbine	9.65	300.1236	-1.89	164.0708, 137.0605	$C_{17}H_{17}NO_4$	(*S*)-2,3,9,10-tetrahydroxyberbine
		11.51	314.1389	-0.69	237.0928, 164.0701, 151.0766, 137.0594	$C_{18}H_{19}NO_4$	Compound **2**
		12.91	328.1535	2.55	219.0830, 178.0857, 151.0772, 119.0509	$C_{19}H_{21}NO_4$	Compound **3**
Cj4'OMT+SiSOMT	(*S*)-2,3,9,10-tetrahydroxyberbine	13.20	328.1532	3.47	192.1023, 164.0704, 137.0592	$C_{19}H_{21}NO_4$	Compound **4**
		17.70	342.169	2.89	178.0860, 151.0747, 119.0509	$C_{20}H_{23}NO_4$	Compound **5**
Cj4'OMT+Cj6OMT	(*S*)-2,3,9,10-tetrahydroxyberbine	9.61	300.1236	-1.89	164.0708, 137.0605	$C_{17}H_{17}NO_4$	(*S*)-2,3,9,10-tetrahydroxyberbine
		15.45	328.1546	-0.81	178.0878, 163.0637, 151.0763, 119.0513	$C_{19}H_{21}NO_4$	Compound **6**
		16.38	328.1540	1.02	178.0879, 163.0637, 151.0763, 119.0504	$C_{19}H_{21}NO_4$	Compound **7**

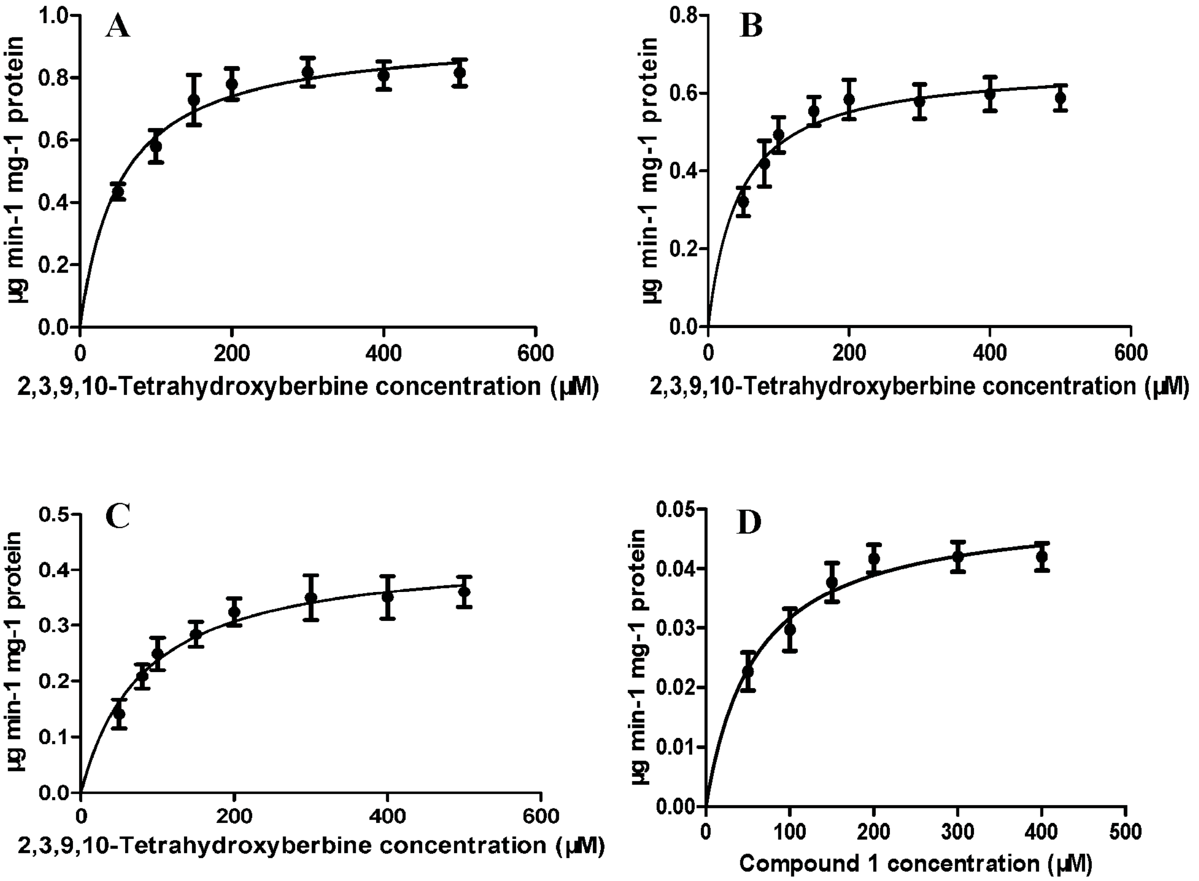

Figure S1 Steady-state kinetics assay of recombinant PsSOMT (A), SiSOMT (B), Cj4'OMT (C), and Cj6OMT (D).

Supporting Information S2

Structural elucidation of compounds

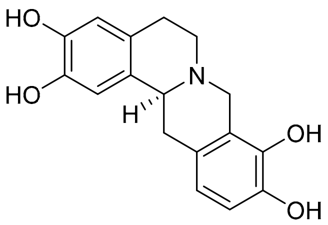

Substrate (2,3,9,10-tetrahydroxyberbine), white powder, $[\alpha]_D^{20}$ -223.76 (*c* 0.28, MeOH), 100% e.e. ^{1}H NMR (600 MHz, MeOD) δ 6.79 (d, *J* = 8.1 Hz, 2H), 6.66 (d, *J* = 12.5 Hz, 2H), 4.72 (d, *J* = 14.0 Hz, 1H), 4.65 (dd, *J* = 11.9, 4.4 Hz, 1H), 4.31 (d, *J* = 14.0 Hz, 1H), 3.87 (d, *J* = 12.5 Hz, 1H), 3.66 (d, *J* = 12.5 Hz, 1H), 3.53 (td, *J* = 11.9, 4.6 Hz, 1H), 3.19 (dd, *J* = 7.6, 5.9 Hz, 1H), 3.02 (m, 1H), 2.93 (d, *J* = 14.5 Hz, 1H). ^{13}C NMR (125 MHz, MeOD) δ 146.95, 146.38, 144.61, 143.16, 123.93, 123.65, 123.28, 120.35, 117.41, 116.44, 116.02, 112.99, 61.72, 53.47, 52.78, 34.28, 26.52.

2,3,9,10-tetrahydroxyberbine ^{1}H NMR

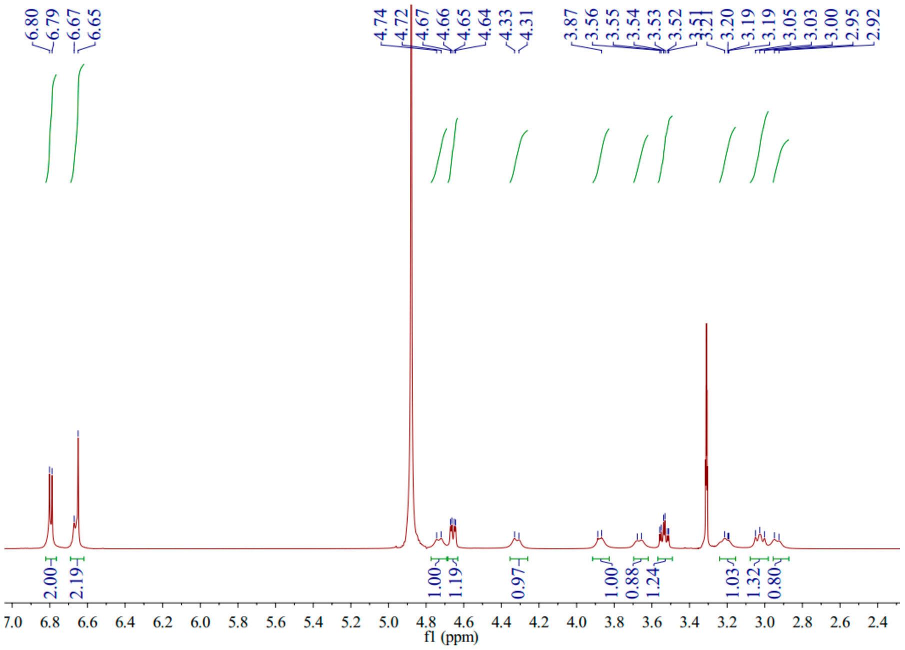

2,3,9,10-tetrahydroxyberbine ^{13}C NMR

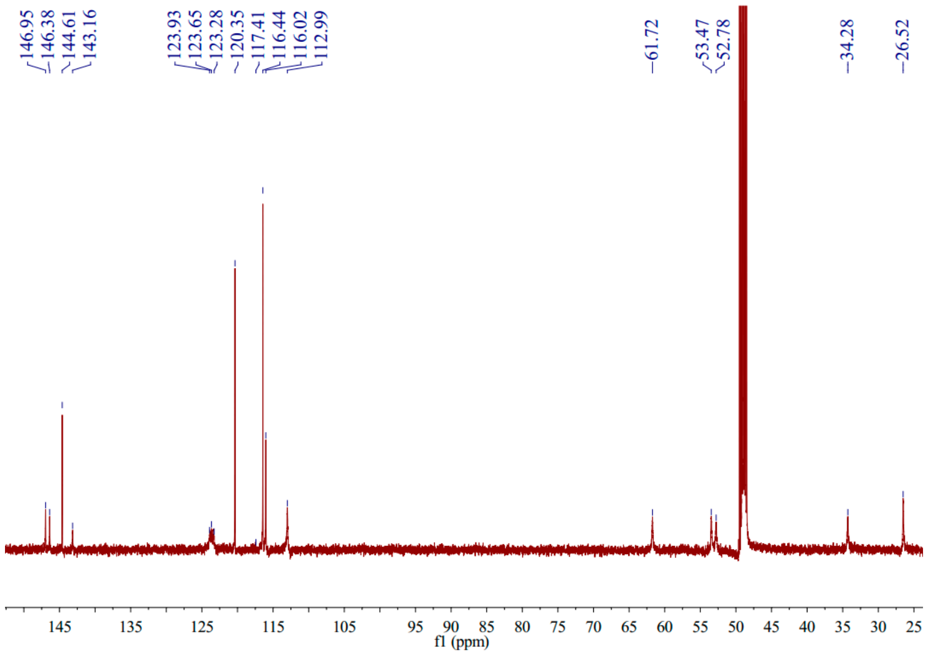

Compound **1** (10-methoxy-2,3,9-tetrahydroxyberbine)

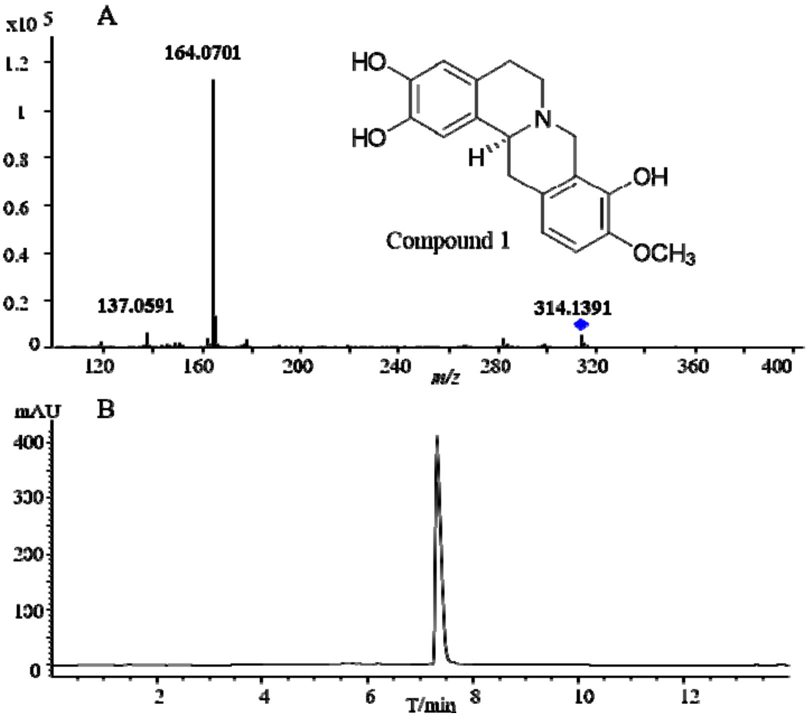

Figure S2 Mass spectrometry and UHPLC of compound **1.** (A) the exact mass of $[M + H]^+$ compound **1**, (B) UHPLC analysis of purified compound **1** at 280 nm.

Compound **1** (10-methoxy-2,3,9-tetrahydroxyberbine), white powder, yield 52 %, $[\alpha]_D^{20}$ -281.90 (*c* 0.12, MeOH), 95.58% e.e. ^{1}H NMR (600 MHz, MeOD) δ 6.80 (1H, d, *J* = 6.0 Hz, H-11), 6.72(1H, s, H-1), 6.65 (1H, d, *J* = 6.0 Hz ,H-12), 6.53 (1H, s, H-4), 4.19 (1H, d, *J* = 15 Hz, H-8), 3.82 (3H, s, H-OMe), 3.50 (1H, m, H-14), 3.44 (1H, dd, *J* = 15 Hz, H-8),3.28(1H, d, *J* = 6Hz, H-13), 3.20 (1H, m, H-6), 3.04 (1H, m, H-5), 2.77 (1H, d, *J* = 6Hz, H-13), 2.63 (2H, m, H-5, H-6).^{13}C NMR (125 MHz, MeOD) δ 146.47, 145.10, 144.97, 143.52, 129.55, 128.49, 126.33, 122.05, 120.04, 115.96, 113.20, 111.18, 60.76, 56.61, 54.80, 52.98, 36.70, 29.04. HRESIMS *m/z*: 314.1391 $[M + H]^+$ (calcd for $C_{18}H_{19}NO_4$, 313.1318).

Compound **1** ^{1}H NMR

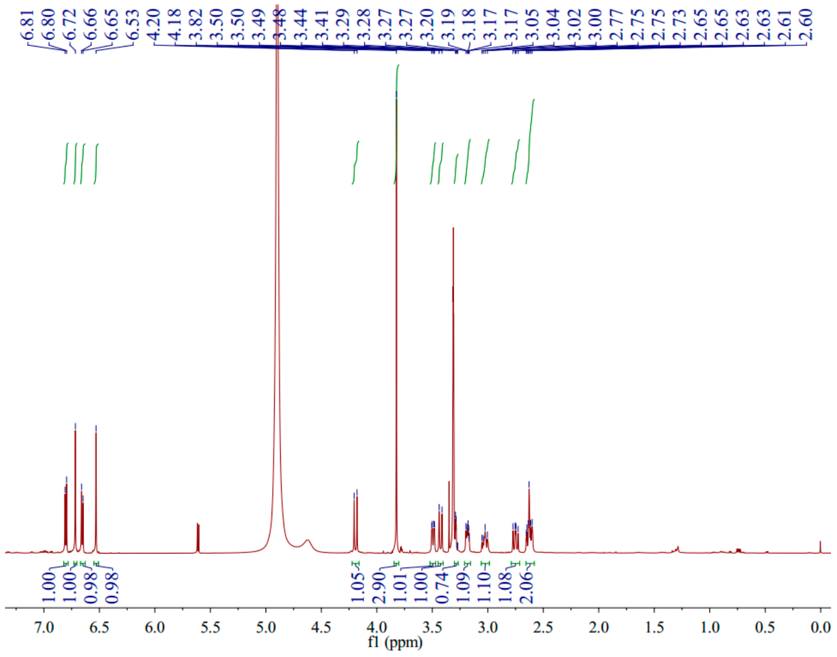

Compound **1** ^{13}C NMR

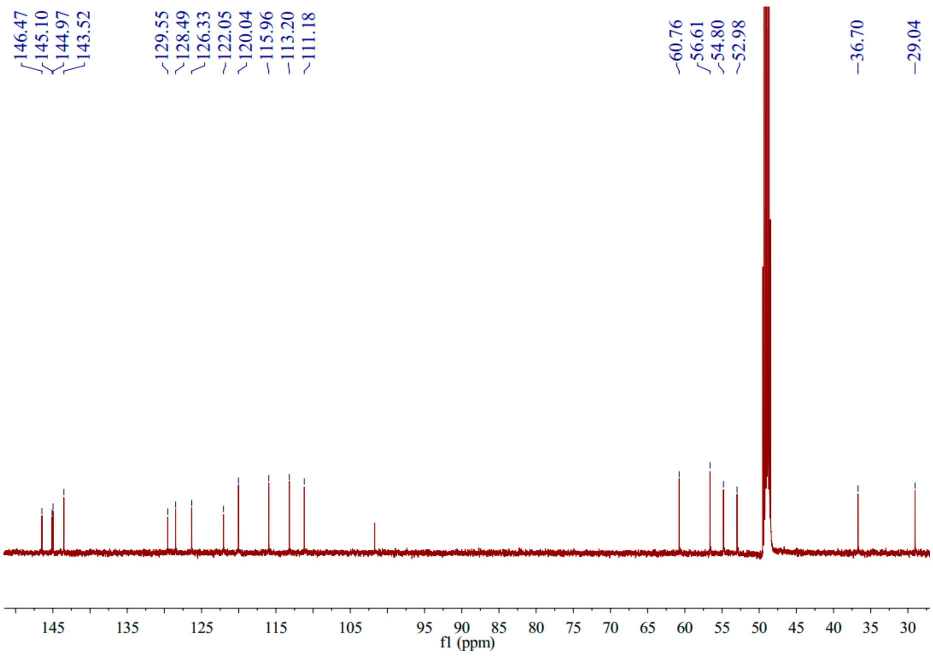

Compound **2** ^{1}H NMR (500 MHz, MeOD) δ 6.82 (1H, d, *J* = 8.3 Hz, H-12), 6.76 (1H, d, *J* = 8.4 Hz, H-11), 6.73 (1H, s, H-1), 6.55 (1H, s, H-4), 4.21 (1H, d, *J* = 15.5 Hz, H-8), 3.83 (3H, s, H-OMe), 3.53 (2H, d, *J* = 15.5 Hz, H-14,H-8), 3.30 (1H, d, *J* = 13.0 Hz, H-13), 3.21 (1H, dd, *J* = 10.0, 8.5 Hz, H-6), 3.05 (1H, t, *J* = 10.0 Hz, H-5), 2.75 (1H, dd, *J* = 13.0, 11.6 Hz, H-13), 2.66 (2H, dd, *J* = 10.0, 8.5 Hz, H-5, H-6). ^{13}C NMR (125 MHz, MeOD) δ 148.82 (C-10), 145.14 (C-3), 145.02 (C-2), 129.52 (C-4a), 128.72 (C-12a), 127.25 (C-8a), 126.27 (C-1a), 125.37 (C-12), 116.40 (C-11), 115.93 (C-4), 113.15 (C-1), 60.85 (C-13a), 60.41 (C-OMe), 54.90 (C-8), 52.98 (C-6), 36.59 (C-13), 29.05 (C-5). HRESIMS *m/z*: 314.1389 [M + H]$^+$ (calcd for $C_{18}H_{19}NO_4$, 313.1316).

Compound **2** ^{1}H NMR

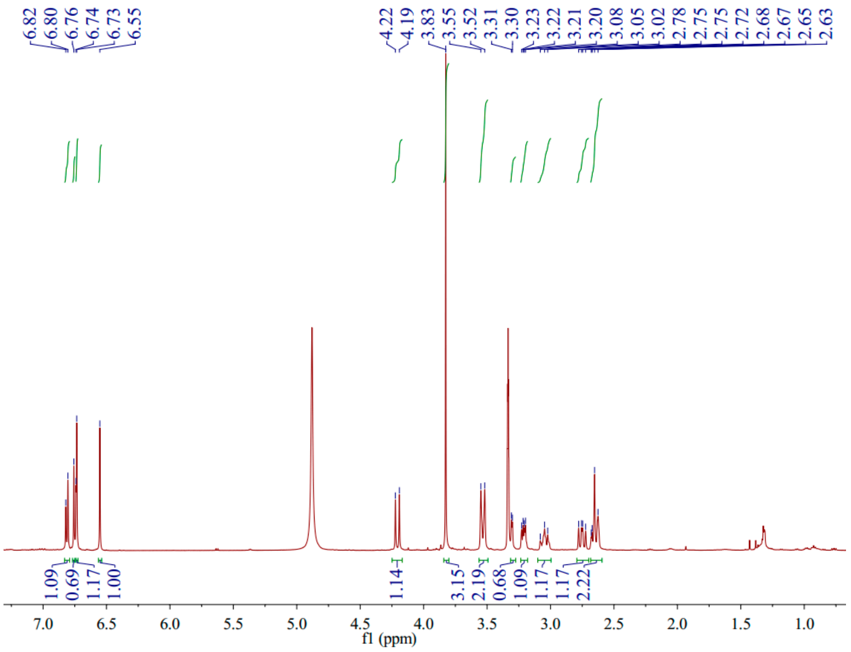

Compound **2** ^{13}C NMR

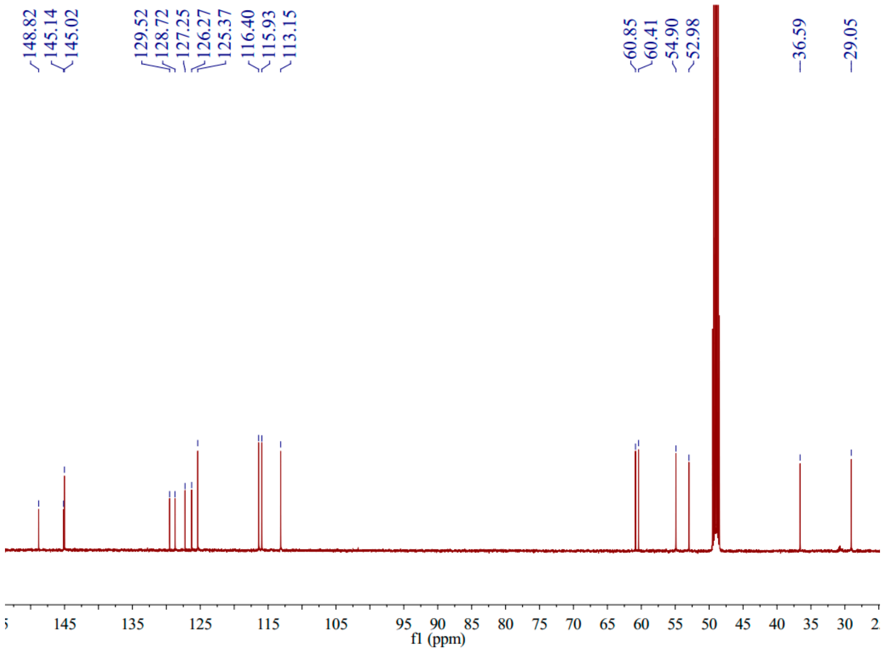

Compound **2** HMQC

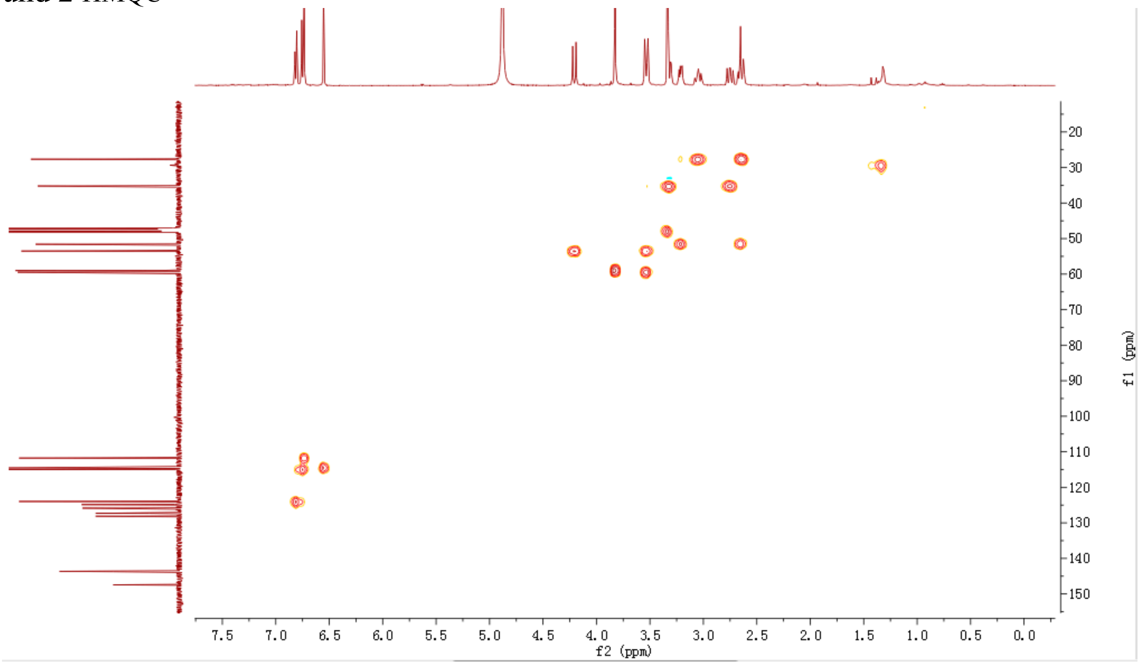

Compound **2** HMBC

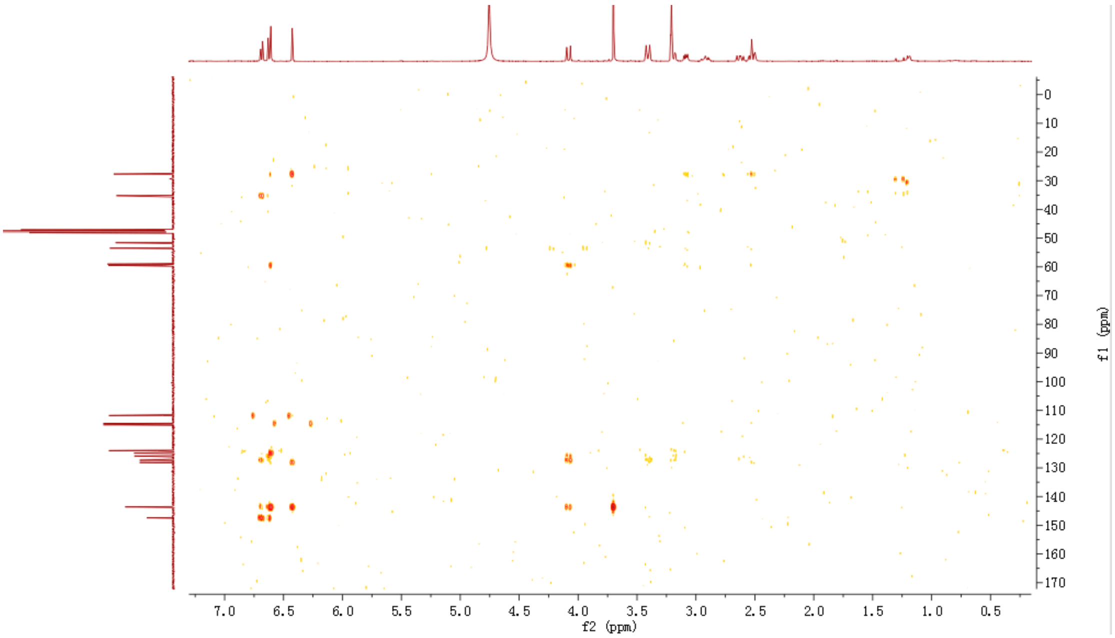

Compound **2** ^{1}H-^{1}HCOSY

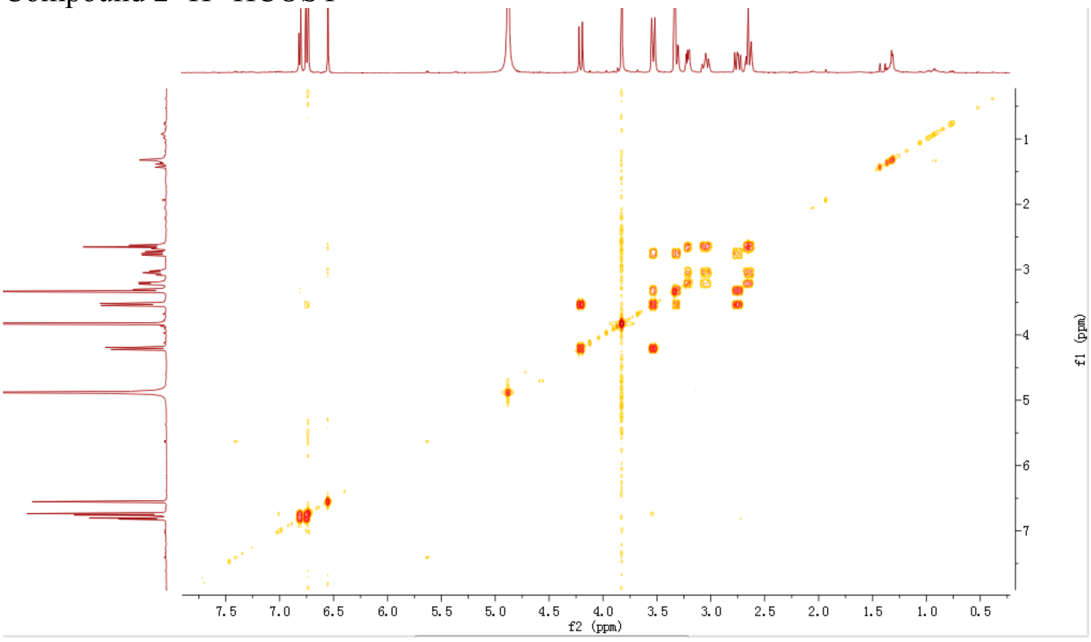

Compound **3** (discretamine) ^{1}H NMR (500 MHz, MeOD) δ 6.84 (1H, s, H-1), 6.82 (1H, d, *J* = 8.3 Hz, H-12), 6.73 (1H, d, *J* = 8.2 Hz,H-11), 6.57 (1H, s, H-4), 4.20 (1H, d, *J* = 15.0 Hz, H-8), 3.85 (3H, s, H-2OMe), 3.81 (3H, s, H-9OMe), 3.59 (1H, dd, *J* = 15.0, 3.6 Hz, H-8), 3.53 (1H, d, *J* = 15.5 Hz, H-14), 3.40 (1H, dd, *J* = 16.0, 11.5 Hz, H-13), 3.20 (1H, d, *J* = 11.0 Hz, H-6), 3.04 (1H, m,H-5), 2.74 (1H, dd, *J* = 16.0, 11.5 Hz, H-13), 2.65 (1H, m, H-5), 2.63 (1H, d, *J* = 11.0 Hz, H-6). ^{13}C NMR (125 MHz, MeOD) δ 148.84 (C-10), 147.89 (C-2), 146.32 (C-3), 145.04 (C-9), 129.48 (C-1a), 128.72 (C-12a), 127.71 (C-4a), 127.28 (C-8a), 125.41 (C-12), 116.41 (C-4), 115.93 (C-11), 110.04 (C-1), 61.01 (C-9OMe), 60.42 (C-13a), 56.61 (C-2OMe), 54.90 (C-8), 52.78 (C-6), 36.58 (C-13), 29.11 (C-5). HRESIMS *m/z*: 328.1535 $[M + H]^+$ (calcd for $C_{19}H_{21}NO_4$, 327.1471).

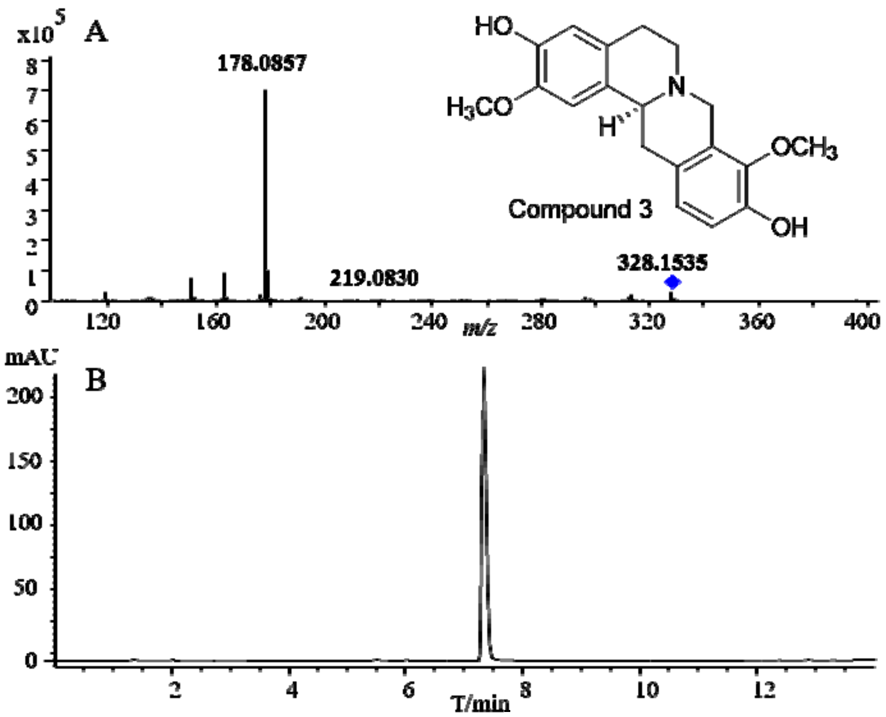

Figure S3 Mass spectrometry and UHPLC of compound **3**. (A) the exact mass of $[M + H]^+$ compound **3**, (B) UHPLC analysis of purified compound **3** at 280 nm.

Compound **3** ^{1}H NMR

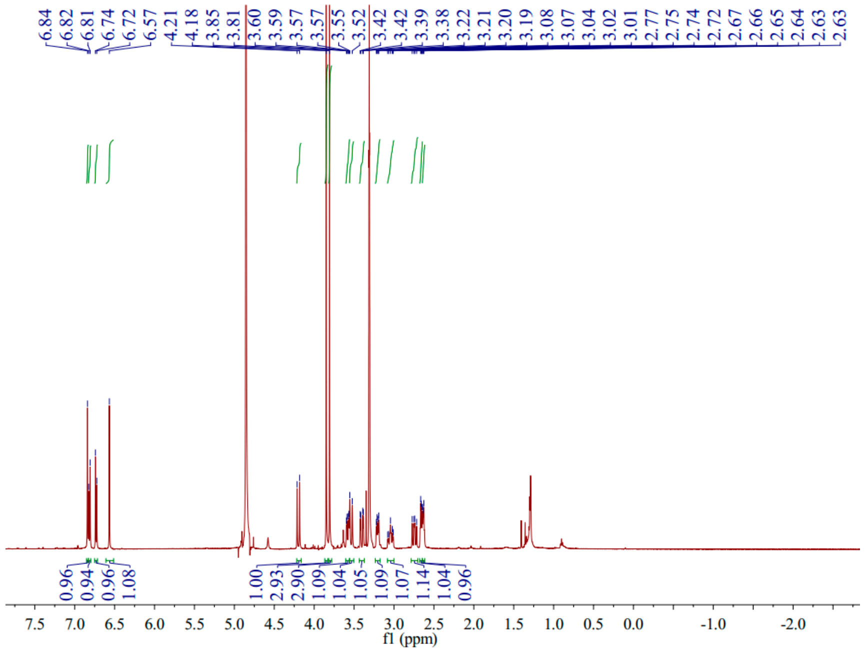

Compound **3** ^{13}C NMR

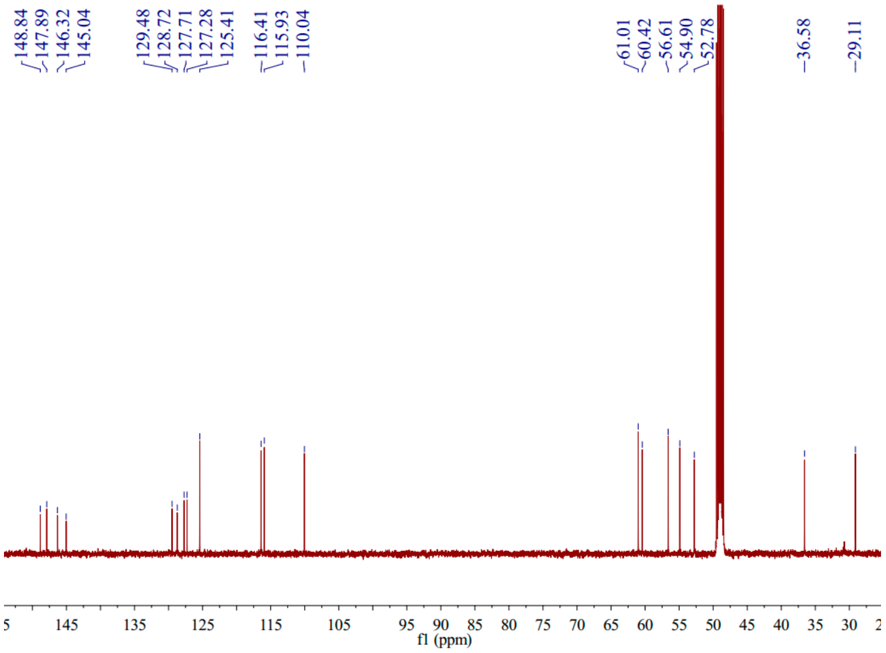

Compound **4** (9,10-methoxy-2,3-tetrahydroxyberbine), white powder, yield 45%, $[\alpha]_D^{20}$ -188.62 (*c* 0.12, MeOH), 98.38% e.e. ^{1}H NMR (600 MHz, $CDCl_3$) δ 6.99 (d, *J* = 12.0 Hz, 2H), 6.75 (s, 1H), 6.59 (s, 1H), 4.51 (d, *J* = 15.5 Hz, 1H), 4.15 (m, 1H), 4.01 (d, *J* = 15.5 Hz, 1H), 3.85 (s, 3H), 3.84 (s, 3H), 3.54 (m, 2H), 3.12 (d, *J* = 8.0 Hz, 2H), 2.92 (dd, *J* = 15.5, 11.9 Hz, 1H), 2.81 (d, *J* = 12.0 Hz, 1H). ^{13}C NMR (150 MHz, MeOD) δ 152.11, 146.32, 146.24, 145.84, 126.56, 126.13, 125.27, 125.20, 124.46, 115.97, 113.67, 113.08, 60.89, 60.73, 56.37, 53.68, 52.37, 34.99, 27.52. HRESIMS *m/z*: 328.1532 [M + H]$^+$ (calcd for $C_{19}H_{21}NO_4$, 327.1459).

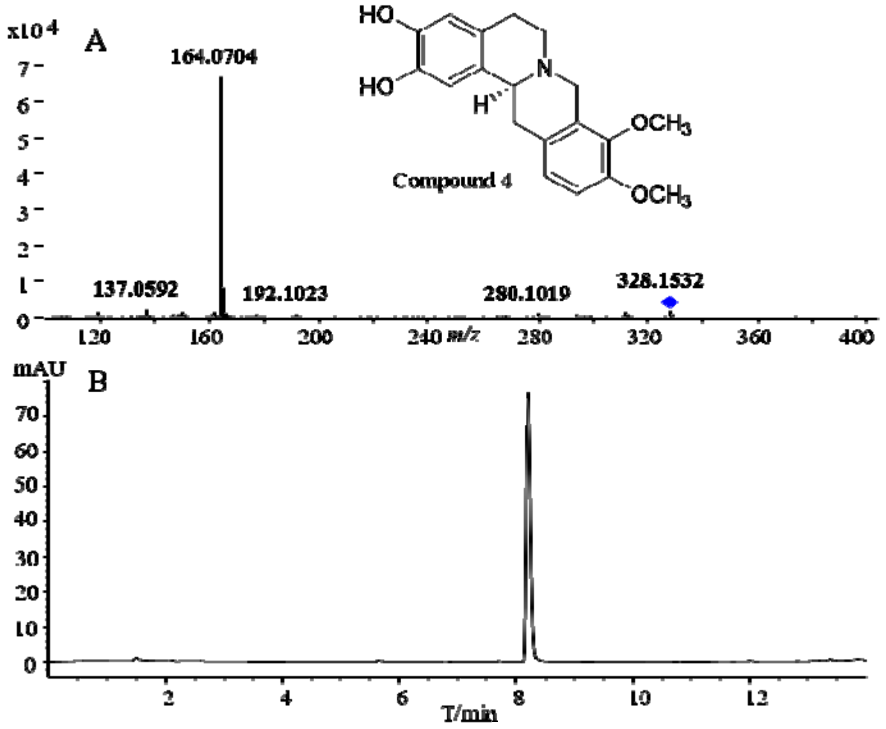

Figure S4 Mass spectrometry and UHPLC of compound **4**. (A) the exact mass of [M + H]$^+$ compound **4**, (B) UHPLC analysis of purified compound **4** at 280 nm.

Compound **4** ^{1}H NMR

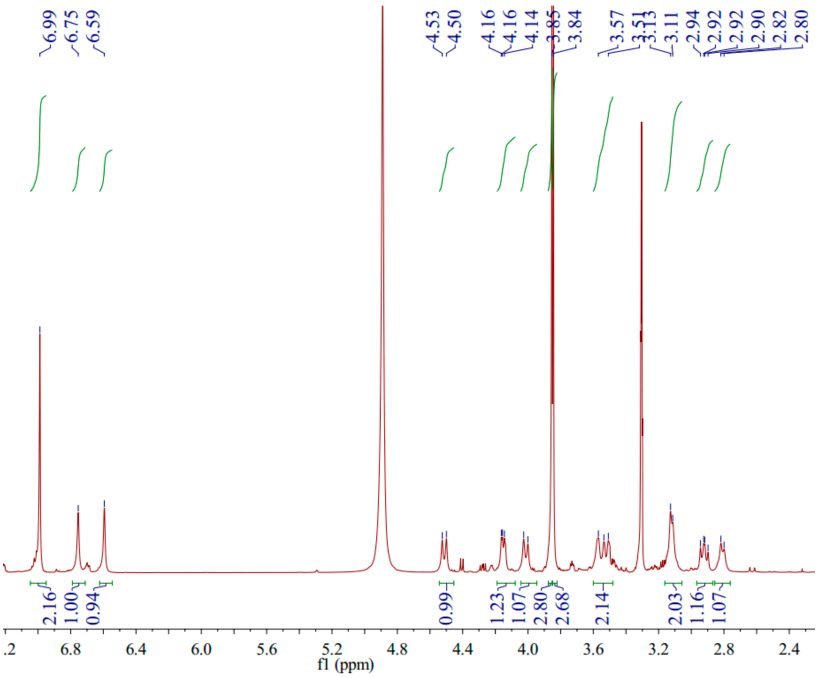

Compound 4 ^{13}C NMR

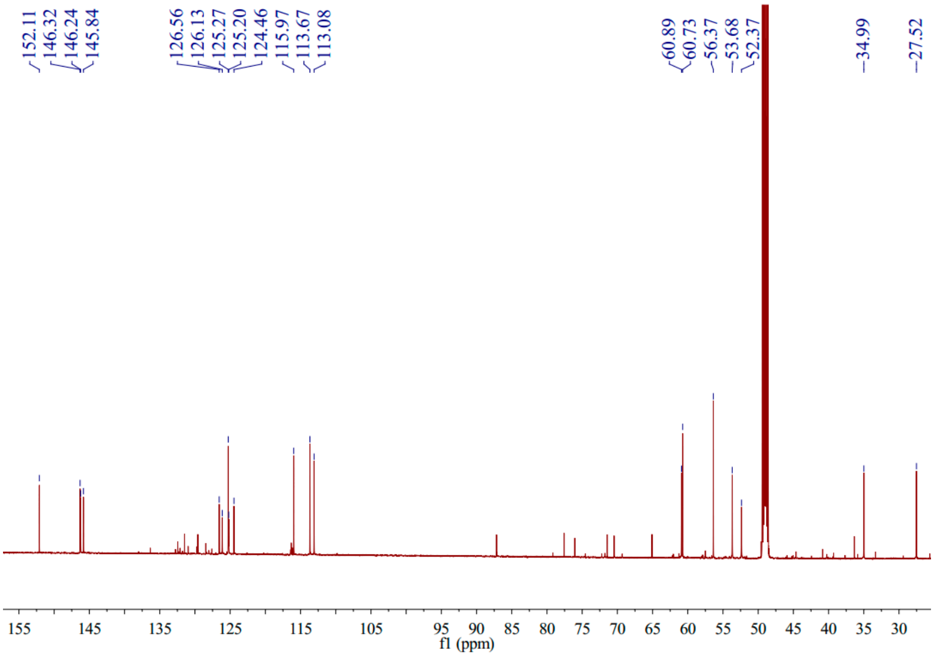

Compound **5** (corypalmine), white powder, yield: 50%, $[\alpha]^{20}_{D}$ -211.67 (*c* 0.12, MeOH), 100% e.e. ^{1}H NMR (600 MHz, $CDCl_3$) δ 6.89 (d, *J* = 8.3 Hz, 1H), 6.80 (d, *J* = 8.3 Hz, 1H), 6.70 (d, *J* = 12.0 Hz, 2H), 4.26 (d, *J* = 12.0 Hz, 1H), 3.90 (s, 3H), 3.85 (d, *J* = 1.0 Hz, 6H), 3.57 (s, 2H), 3.23 (m, 3H), 2.86 (s, 1H), 2.66 (d, *J* = 11.1 Hz, 2H). ^{13}C NMR (150 MHz, $CDCl_3$) δ150.45, 145.30, 145.21, 144.15, 129.42, 128.77, 127.76, 127.58, 123.97, 114.37, 111.10, 107.87, 60.34, 59.52, 56.20, 56.01, 54.09, 51.63, 36.48, 28.94. HRESIMS: *m/z* 342.1690 [M + H]$^{+}$ (calcd for $C_{20}H_{23}NO_4$, 341.1617).

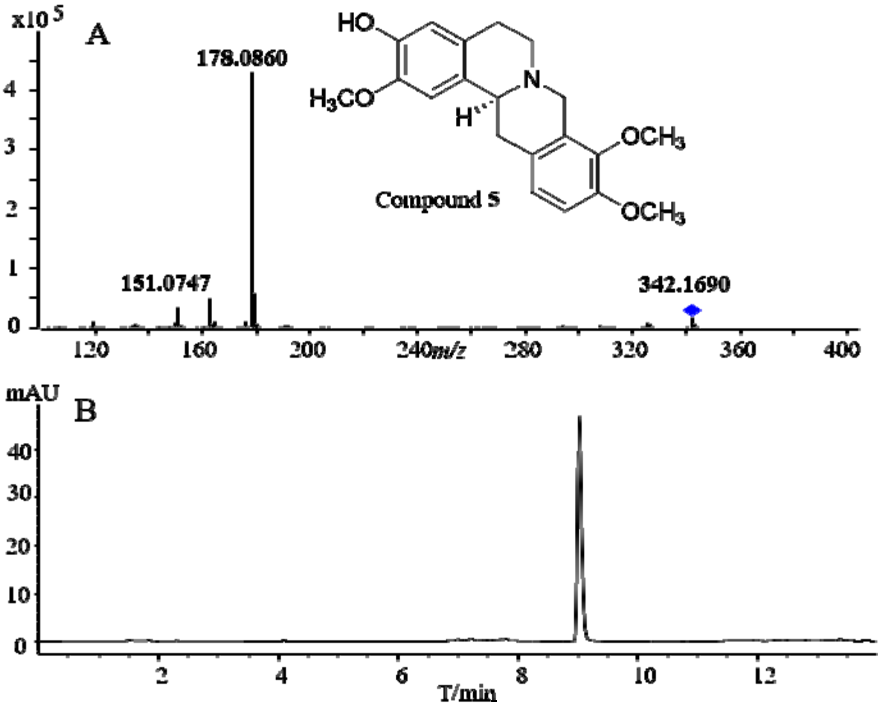

Figure S5 Mass spectrometry and UHPLC of compound **5**. (A) the exact mass of [M + H]$^{+}$ compound **5**, (B) UHPLC analysis of purified compound **5** at 280 nm.

Compound **5** ^{1}H NMR

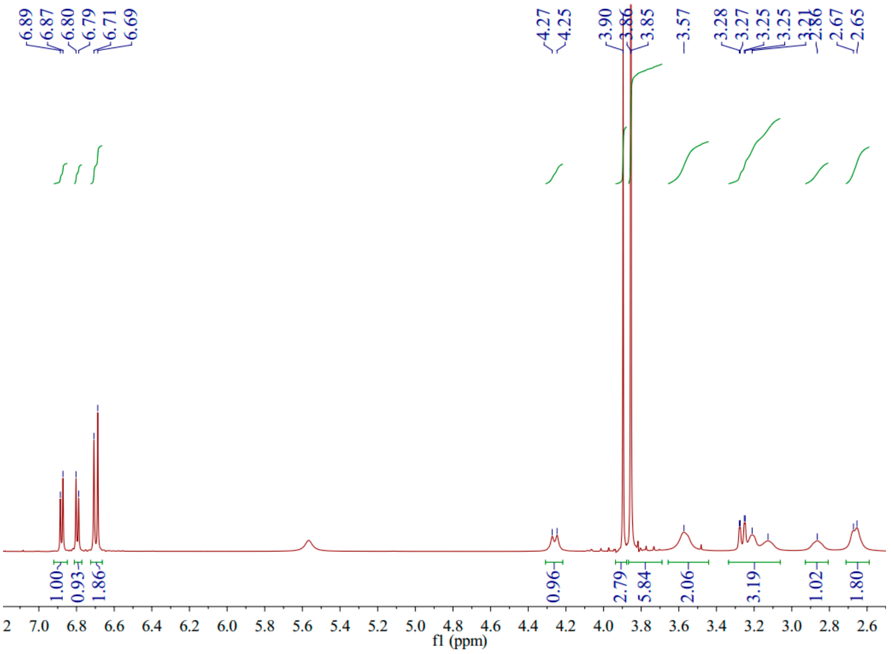

Compound **5** ^{13}C NMR

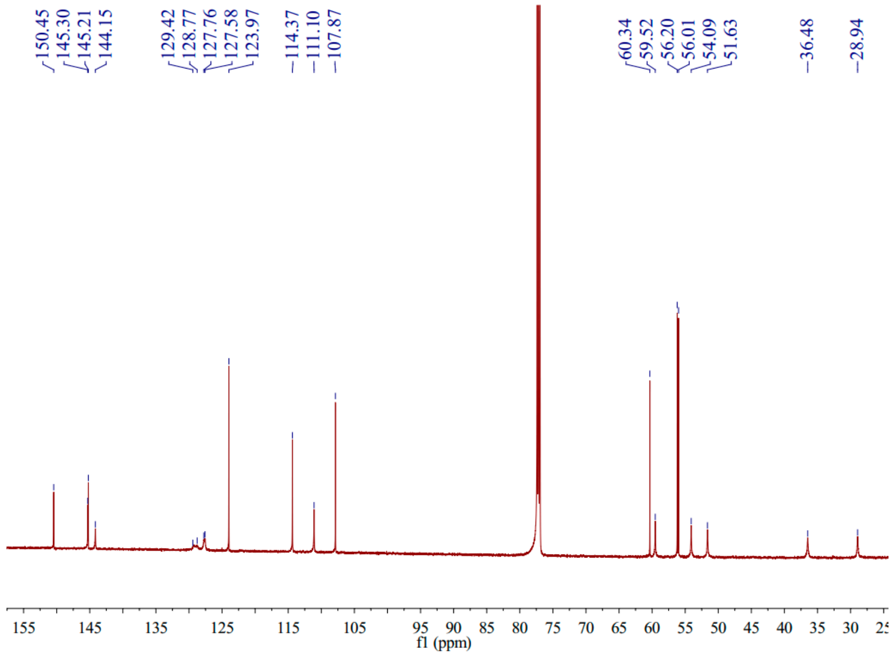
150.45
145.30
145.21
144.15
129.42
128.77
127.76
127.58
123.97
114.37
111.10
107.87
60.34
59.52
56.20
56.01
54.09
51.63
36.48
28.94
155
145
135
125
115
105
95
90
85
80
75
70
65
60
55
50
45
40
35
30
f1 (ppm)

Compound **6** (scoulerine), yellow powder, yield 28%, $[\alpha]_D^{20}$ -220.26 (*c* 0.12, MeOH), 100% e.e. ^{1}H NMR (600 MHz, MeOD) δ 6.80 (d, *J* = 10 Hz, 1H), 6.75 (s, 1H), 6.66 (m, 2H), 4.19 (d, *J* = 10 Hz, 1H), 3.82(s, 6H), 3.49(d, *J* = 10 Hz, 1H), 3.42(d, *J* = 15 Hz, 1H), 3.29 (m, 1H), 3.15(dd, *J* = 5Hz, 10Hz, 1H), 3.08 (m, 1H), 2.74 (m, 1H), 2.68 (m, 1H), 2.63 (m, 1H). ^{13}C NMR (150 MHz, MeOD) δ 147.84, 146.46, 146.04, 143.53, 130.82, 128.48, 126.33, 122.14, 120.02, 113.09, 112.49, 111.09, 60.71, 56.55, 56.32, 54.85, 52.96, 36.70, 29.31. HRESIMS m/z 328.1546 $[M + H]^+$ (calcd for $C_{19}H_{21}NO_4$, 327.1473)

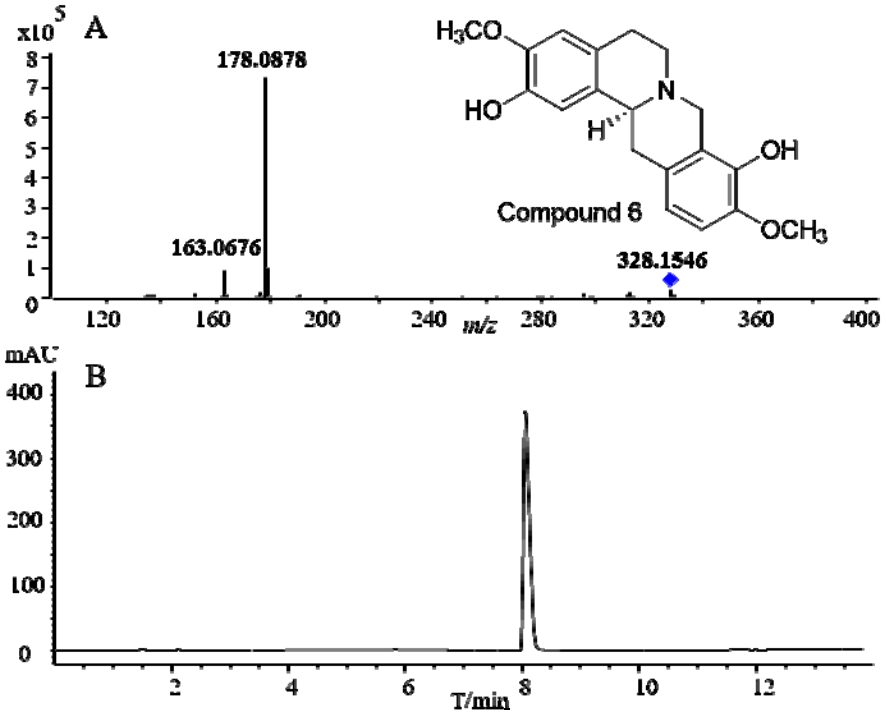

Figure S6 Mass spectrometry and UHPLC of compound **6**. (A) the exact mass of $[M + H]^+$ compound **6**, (B) UHPLC analysis of purified compound **6** at 280 nm.

Compound **6** ^{1}H NMR

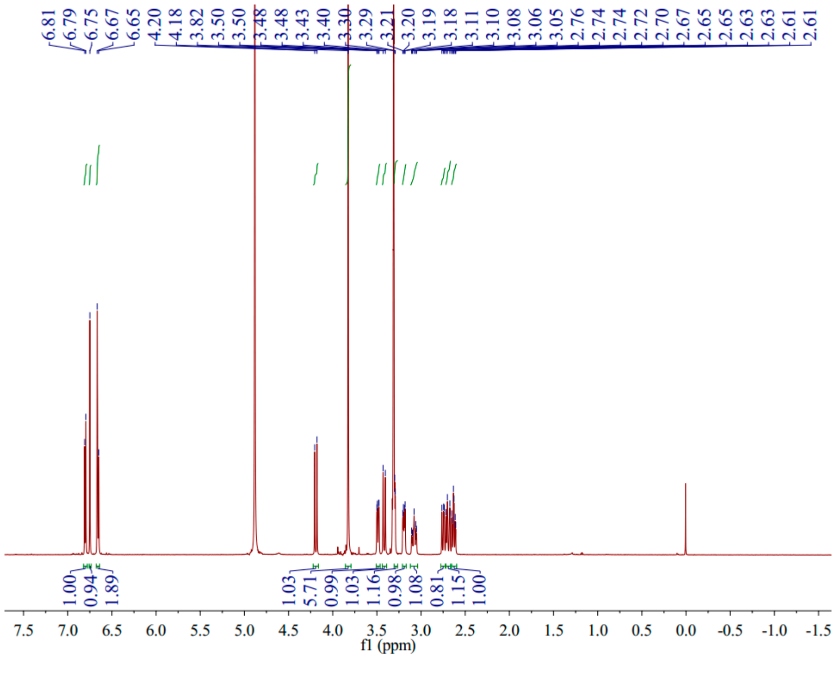

Compound **6** ^{13}C NMR

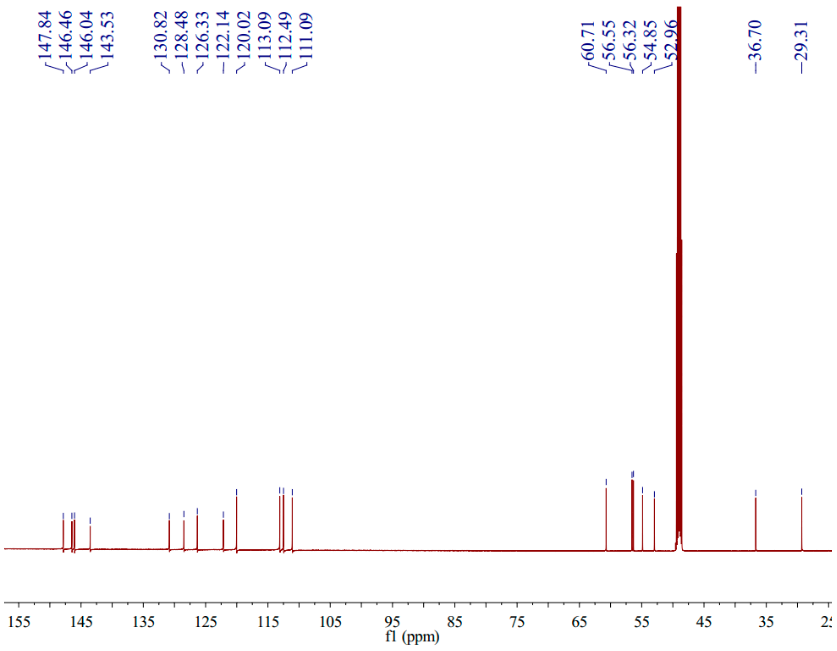

Compound **7** (isoscoulerine), yellow powder, yield 61%, $[\alpha]_D^{20}$ -223.57 (*c* 0.16, MeOH), 100% e.e. ^{1}H NMR (500 MHz, MeOD) δ 6.91 (m, 2H), 6.77 (d, *J* = 10 Hz, 1H), 6.65 (s, 1H), 4.57 (d, *J* = 15 Hz, 1H), 4.33 (d, *J* = 10Hz, 1H), 4.06 (d, *J* = 15Hz, 1H), 3.87(d, *J* = 15Hz, 6H), 3.66 (m, 2H), 3.23 (m, 1H), 3.16 (m, 1H), 2.95 (m, 1H), 2.87 (d, *J* = 15Hz, 1H).^{13}C NMR (125 MHz, MeOD) δ 148.59, 147.51, 147.04, 143.98, 132.31, 126.05, 125.62, 120.18, 117.79, 116.02, 112.24, 109.85, 61.15, 56.63, 56.58, 53.54, 52.01, 34.80, 27.26. HRESIMS *m/z*: 328.1540 $[M + H]^+$ (calcd for $C_{19}H_{21}NO_4$, 327.1467).

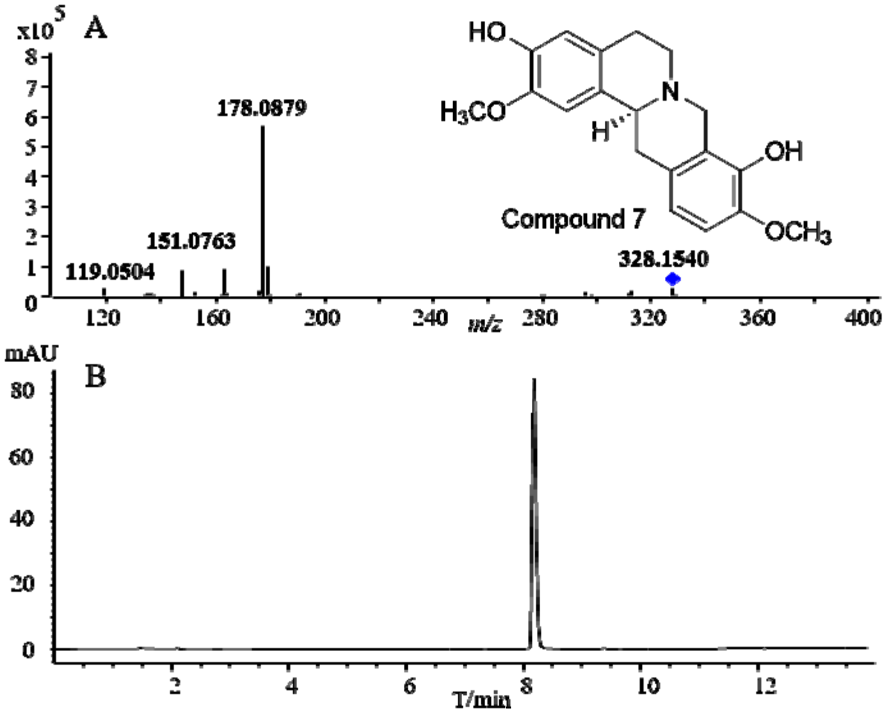

Figure S7 Mass spectrometry and UHPLC of compound **7**. (A) the exact mass of $[M + H]^+$ compound **7**, (B) UHPLC analysis of purified compound **7** at 280 nm.

Compound **7** ^{1}H NMR

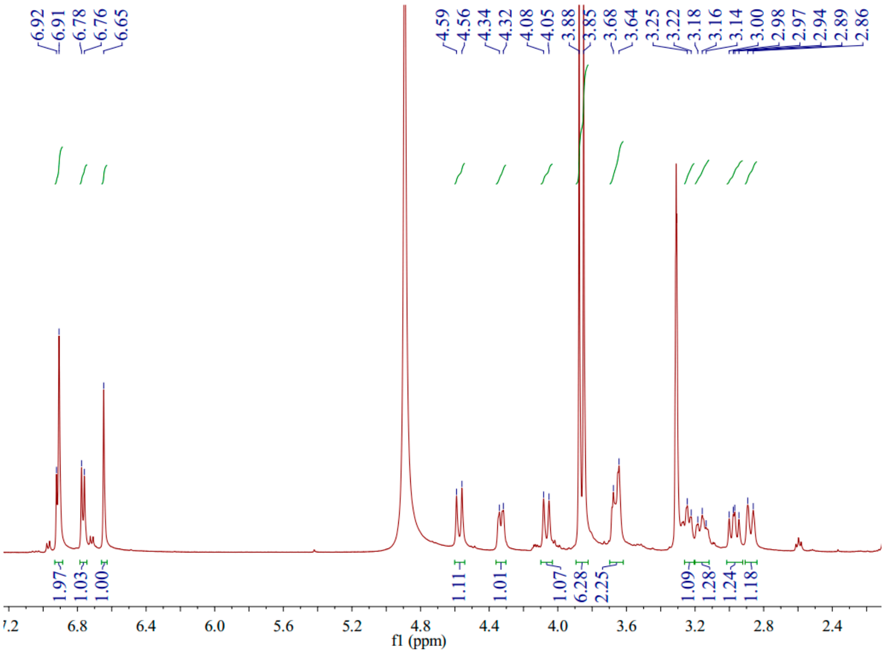

Compound **7** ^{13}C NMR

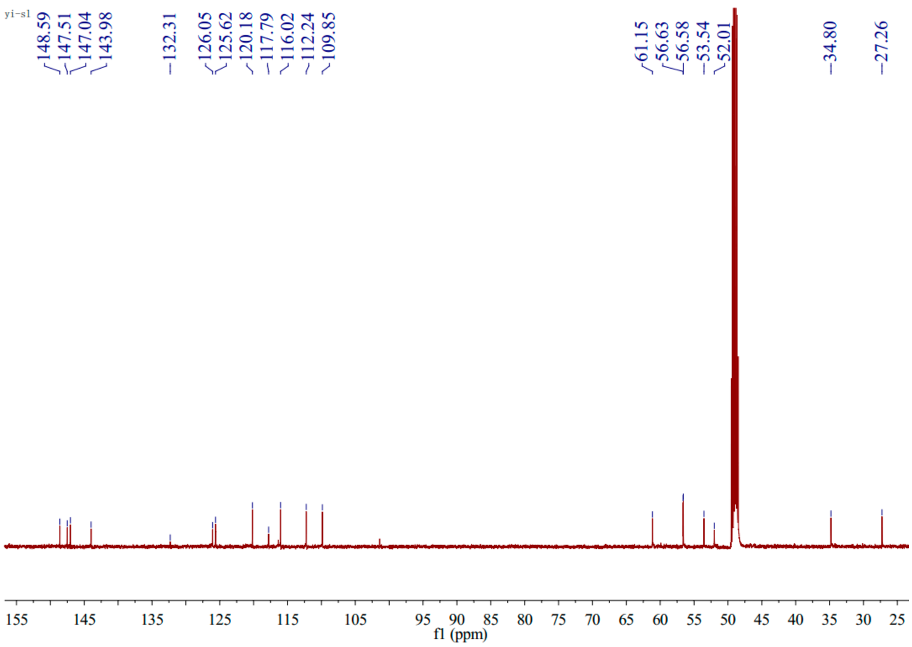

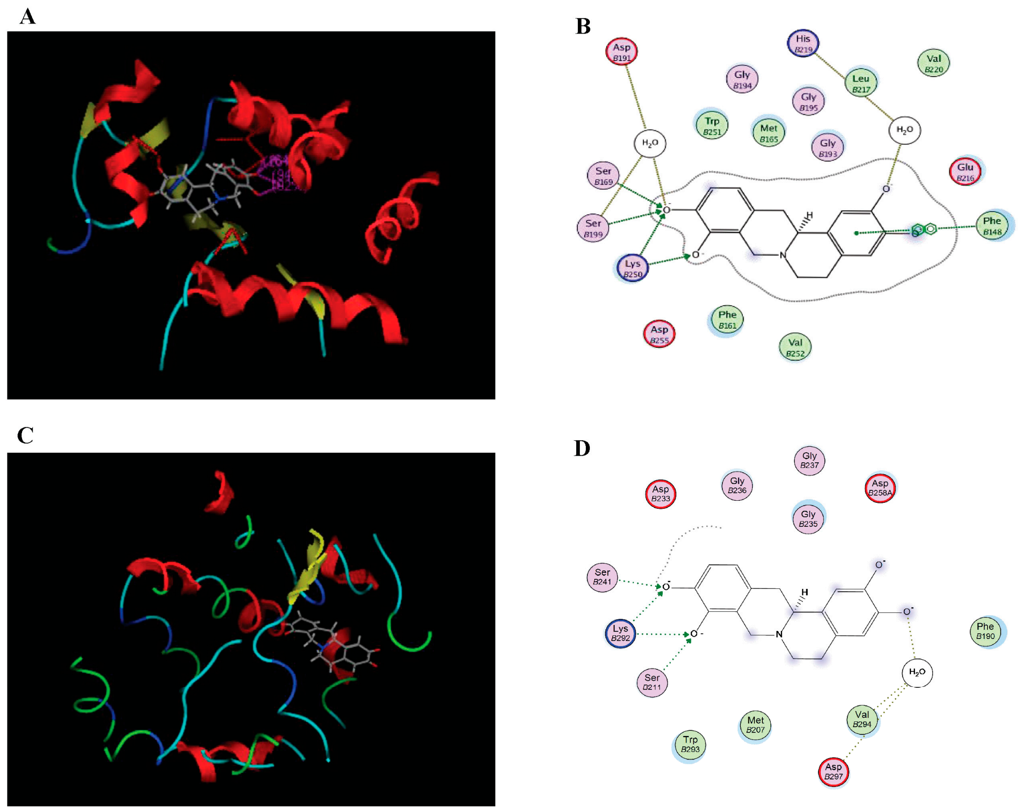

Figure S8 Homology modeling and molecular docking of SiSOMT, PsSOMT to the substrate. A: the substrate (thick grey lines) in the ligand binding domain of SiSOMT (ribbon representation). B: The interaction between substrate and the amino acid residues from the active site of SiSOMT. The proximity contour (dashed lines) and solvent exposed areas (solid purple spheres) of the ligand atoms are indicated, as are the polar (pink), hydrophobic (green), and solvent-exposed (light blue shadow) binding pocket amino acids. Acidic and basic residues are highlighted with red and blue halos, respectively; C: the substrate in the ligand binding domain of PsSOMT. D: The interaction between substrate and amino acid residues from the active site of PsSOMT.
